# OPSALC: On-Particle Solvent-Assisted Lipid Coating to Create Erythrocyte Membrane-like Coatings with Improved Hemocompatibility

**DOI:** 10.1021/acsami.5c02103

**Published:** 2025-03-13

**Authors:** Francisca L. Gomes, Dorothee Wasserberg, Rick Edelbroek, Jasper van Weerd, Pascal Jonkheijm, Jeroen Leijten

**Affiliations:** † Department of Bioengineering Technologies, Leijten Laboratory, Faculty of Science and Technology, Technical Medical Centre, University of Twente, Drienerlolaan 5, Enschede 7522NB, The Netherlands; ‡ Department of Molecules and Materials, Laboratory of Biointerface Chemistry, Faculty of Science and Technology, Technical Medical Centre and MESA+ Institute, University of Twente, Drienerlolaan 5, Enschede 7522NB, The Netherlands; § LipoCoat BV, Hengelosestraat 535, Enschede 7521AG, The Netherlands

**Keywords:** lipids, membranes, particles, micromaterials, hemocompatibility, coatings

## Abstract

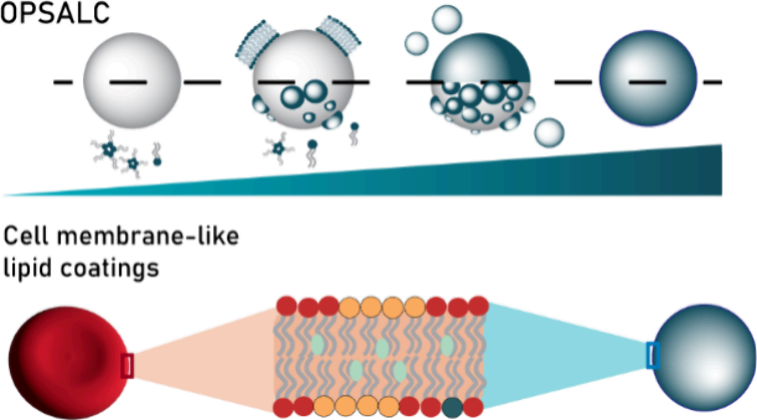

Particles are essential building blocks in nanomedicine and cell engineering. Their administration often involves blood contact, which demands a hemocompatible material profile. Coating particles with isolated cell membranes is a common strategy to improve hemocompatibility, but this solution is nonscalable and potentially immunogenic. Cell membrane-like lipid coatings are a promising alternative, as lipids can be synthesized on a large scale and used to create safe cell membrane-like supported bilayers. However, a method to controllably and scalably lipid-coat a wide range of particles has remained elusive. Here, an on-particle solvent-assisted lipid coating (OPSALC) method is introduced as an innovative technique to endow various types of particles with cell membrane-like coatings. Coating formation efficiency is shown to depend on lipid concentration, buffer addition rate, and solvent:buffer ratio, as these parameters determine lipid assembly and lipid–surface interactions. Four lipid formulations with various levels of erythrocyte membrane mimicry are explored in terms of hemocompatibility, demonstrating a reduced particle-induced hemolysis and plasma coagulation time. Interestingly, formulations with higher mimicry levels show the lowest levels of complement activation and highest colloidal stability. Overall, OPSALC represents a simple yet scalable strategy to endow particles with cell membrane-like lipid coatings to facilitate blood-contact applications.

## 1 Introduction

Particles are an essential foundation of nanomedicine, having contributed to the establishment of targeted drug delivery, enhanced imaging, and precision diagnostics.^1−5^ Particles have recently started being explored as cell-like materials, with emerging reports on engineered platelets,^6^ macrophages^7,8^ and red blood cells (erythrocytes)^9−11^ for targeted therapeutics. Particle administration is often done via intravenous or intratumoral delivery,^2,5^ which includes blood contact, and therefore demands the use of hemocompatible materials.

Most particles (e.g., micro and nanomaterials) are either poorly or not fully hemocompatible, and demand surface modifications or coatings to facilitate their use in blood-contact applications. Common strategies to improve particle hemocompatibility include the application of surface modifications or coatings, such as heparin,^12−14^ albumin^14−16^ or lipids,^17−20^ but the use of heparin poses some risk of thrombotic events in patients^21,22^ and the use of albumin coatings shows a short-lasting effect, predominantly due to the displacement of albumin by other serum proteins in blood.^21^ Recently, the use of blood cell lipid membrane “ghosts”, i.e. membranes isolated from leukocytes, platelets, or erythrocytes, has shown great promise in the improvement of particle hemocompatibility, demonstrating a notable reduction in macrophage response and prolongation of circulation time.^23−27^ Despite their advantages, blood cell membrane “ghosts” are scarcely available, contain inherently instable membrane proteins,^28^ and may give rise to patient-specific complications due to the immunogenicity of membrane antigens. To overcome these obstacles, artificial lipid membrane coatings can be engineered using synthetic lipids, ultimately providing a stable, tunable, and disease-free coating.^29,30^ However, an efficient and scalable method to apply cell membrane-like lipid nanocoatings on particles remains unexplored.

Lipid coatings can be applied to material surfaces through a number of techniques, such as vesicle fusion,^31−33^ Langmuir–Blodgett deposition^34,35^ or dip pen lithography.^36^ One of the most commonly employed techniques in the lipid-coating of particles is vesicle fusion, wherein small uniform lipid vesicles adhere to a particle surface, rupture and spread out to form a continuous bilayer. This process has been studied in great detail for forming supported lipid bilayers on planar surfaces.^37−41^ Similarly, to efficiently coat particles with lipids through vesicle fusion, the pH, ionic strength and the choice of charged lipid headgroups need optimization to enhance vesicle absorption to the surface due to attractive forces, while reducing intervesicle interactions and particle aggregation.^33^ Consequently, for most particle material-lipid combinations, lipid vesicles do not adsorb to particles or, when they do adsorb, they often only partially rupture at the surface, yielding incompletely covered particles.^40,42^ To overcome these obstacles, exciting advances have been reported on solvent-exchange protocols that allow for the lipid-coating of a near-universal variety of planar solid surfaces.^43−46^ In these solvent-exchange protocols, the organic solvent, in which the lipids are dissolved, is gradually diluted by the addition of water. This solvent–water gradient will gradually induce a lipid assembly from monomeric lipids and inverted micelles to micelles, and then vesicles.^47^ Shortly before the onset of the micelle-to-vesicle transition, a lipid bilayer nucleates at the solid planar surface, forming a cell membrane-like lipid layer coating.^47^ In contrast to vesicle fusion, solvent-exchange protocols require less handling and can be easily implemented in different scalable formats such as continuous flow set-ups. In addition, particles would not require a laborious tailoring of their surface prior to applying solvent-exchange.^43,47^ While these strategies have been vastly successful in applying lipid coatings to planar substrates, the successful lipid-coating of nonplanar substrates, such as particles, through solvent-exchange methods has not yet been documented.

**Figure 1:**
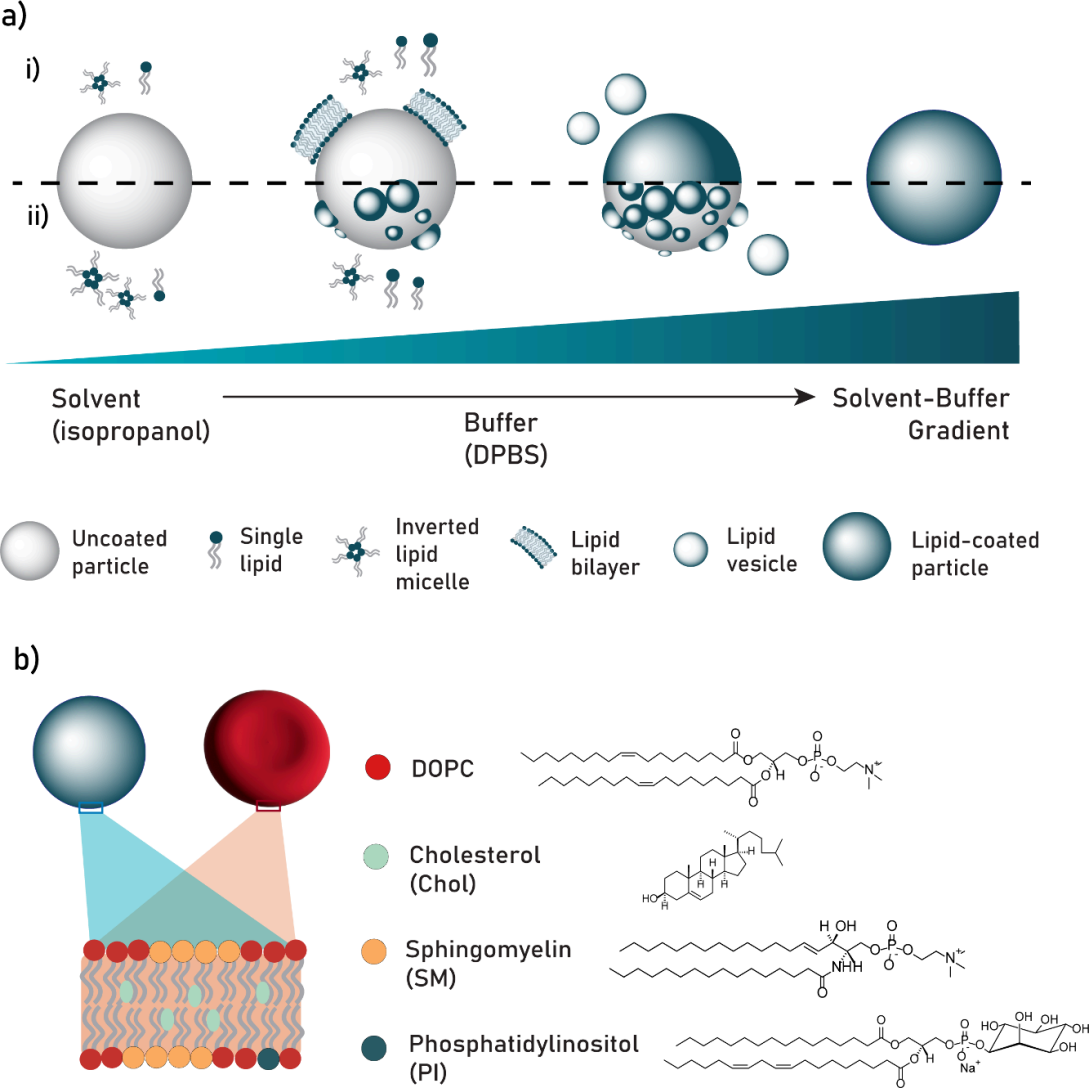
(a) Schematic depiction of the process of on-particle solvent-assisted lipid coating (OPSALC), including the different possible steps of lipid layer self-assembly, which are discussed in the text, involving (i) lipid monomers and small lipid assemblies and (ii) vesicle fusion. (b) Schematic depiction of an erythrocyte membrane-like lipid coating. Chemical structures of the lipids used in this study.

In this work, we introduce an on-particle solvent-assisted lipid coating (OPSALC) method, which combines the versatility of solvent exchange lipid coating methods with a simple experimental setup, compatible with a wide range of particle substrates. In short, an aqueous buffer is gradually added, while stirring, to a concentrated mixture of lipids and particles in a water-miscible alcohol. The induced gradient then originates a lipid-layer coating on the particles through a gradual lipid assembly (Figure 1a, Figure S1). We first validated the method using a single-lipid solution of 1,2-dioleoyl-*sn*-glycero-3-phosphocholine (DOPC) on silica nanoparticles (SiO_2_ NPs) and microparticles (SiO_2_ MPs). Next, we applied the OPSALC method to coat SiO_2_ and polycaprolactone (PCL) particles using four erythrocyte cell membrane-mimicking lipid coatings (Figure 1b) to study their influence on the overall hemocompatibility of the particles.

## 2 Experimental Section

### 2.1 Materials

Colloidal silica nanoparticles (diameter 0.10 ± 0.03 μm) were purchased from Polysciences Inc. DiagNano green fluorescent silica microparticles (diameter 5 μm), monodisperse, with hydroxyl surface functionalization, were purchased from CD Bioparticles. Polycaprolactone (PCL) M_*w*_ ∼ 14,000, Mowiol 40–88, HEPES (≥99.5%), calcium chloride dihydrate (ACS reagent, ≥ 99.0%), sodium chloride (ACS reagent, ≥ 99.0%) and ethylenediamine-tetraacetic acid disodium salt dihydrate (EDTA, ≥ 99.0%) were purchased from Sigma-Aldrich. 1,2-dioleoyl-*sn*-glycero-3-phosphocholine (DOPC), cholesterol, egg sphingomyelin (SM), and soy phosphatidylinositol (PI) were purchased from Avanti Polar Lipids (Sigma). Texas Red-DHPE (TR-DHPE) was purchased from ThermoFisher Scientific. All lipids were purchased in powder form, dissolved in chloroform, and stored at −20 °C except TR-DHPE, which was solubilized in methanol and then stored at −20 °C. Dulbecco’s Phosphate Buffered Saline (DPBS, sterile), DPBS with CaCl_2_ and MgCl_2_ (sterile), chloroform (ACS spectrophotometric grade, ≥ 99.8%), dichloromethane (DCM, ACS reagent, ≥ 99.5%), methanol (HPLC grade, ≥ 99.9%), and isopropanol (IPA, ACS reagent, ≥ 99.5%) were purchased from Sigma-Aldrich. Ethanol (absolute, ≥ 99.5%) was purchased from VWR Chemicals. Ultrapure water (resistivity 18.2 MΩ•cm) was obtained from a Milli-Q setup (Millipore). DPBS was sterile-filtered with 0.2 μm syringe filters prior to each experiment.

### 2.2 OPSALC on SiO_2_ Nanoparticles

Suspensions of SiO_2_ NPs were prepared in ethanol and isopropanol at a concentration of 10 mg mL^–1^. A lipid solution of DOPC was prepared in the same solvents at concentrations of 0.5, 1.0, 2.0, and 4.0 mg mL^–1^. To coat the particles through the OPSALC method, a concentrated lipid-particle suspension was first prepared by adding 50 μL of SiO_2_ NPs to 450 μL of lipid solution in a small glass vial. The gradient was then induced by the injection of DPBS into the vial at a specific addition rate (600 μL min^–1^ or 60 μL min^–1^) using a syringe pump (KD Scientific). This buffer addition was performed under magnetic stirring at 500 rpm, up to a volume of 5 mL, reaching a final solvent:buffer ratio of 10:90 (v/v). The resulting suspension was then heated to 50 °C for 45 min and left to cool down to room temperature. Particles were washed three times in ultrapure water by centrifuging at 10000 *g* for 10 min at 4 °C. During washing, the supernatant was removed and the pellet was resuspended in fresh ultrapure water using a micropipette (Eppendorf). After washing, particle pellets were kept at 4 °C until further use.

For the study on the effect of lipid concentration, the lipid-particle mixture was prepared in ethanol using a buffer addition rate of 600 μL min^–1^ and a lipid concentration of 0.5, 1.0, 2.0, or 4.0 mg mL^–1^, corresponding to an approximate lipid bilayer:particle ratio (*n*) of 4:1, 8:1, 16:1, and 32:1 (Methods S1). For the study on buffer addition rate, the lipid-particle mixture was prepared in ethanol using a lipid concentration of 1.0 mg mL^–1^ and a buffer addition rate of 600 or 60 μL min^–1^. Lipid vesicles were produced as the respective controls, under the same conditions, using pure ethanol instead of the SiO_2_ NP suspension. For the study on solvent, the lipid-particle mixture was prepared in ethanol or isopropanol using a lipid concentration of 1.0 mg mL^–1^ and a buffer addition rate of 600 μL min^–1^. Lipid vesicles were produced as the respective controls, under the same conditions, using pure ethanol or isopropanol instead of the SiO_2_ NP suspension. Lastly, for the study on solvent:buffer ratio, the lipid-particle mixture was prepared in ethanol using a lipid concentration of 1.0 mg mL^–1^ and an addition rate of 600 μL min^–1^, at a final solvent:buffer ratio of 100:0, 50:50, or 10:90 (v/v). The final volume was maintained at 5 mL, and the ratios were obtained by adding 4.5 mL of ethanol (100:0), 2 mL of ethanol with 2.5 mL of DPBS (50:50), or 4.5 mL of DPBS (10:90).

### 2.3 OPSALC on SiO_2_ Microparticles

Suspensions of SiO_2_ MPs were prepared in ethanol and isopropanol at a concentration of 10 mg mL^–1^. A fluorescent lipid solution of DOPC (99.9 mol % DOPC, 0.1 mol % TR-DHPE) was prepared in the same solvents at concentrations of 0.25, 0.5, 1.0, and 2.0 mg mL^–1^_,_ corresponding to 0.1, 0.2, 0.4, and 0.8 mg per mg of SiO_2_ MPs, or *n* ≈ 44, 88, 176, and 353 lipid bilayers per particle, respectively (Methods S2). To coat the particles through the OPSALC method, a concentrated lipid-particle suspension was prepared by mixing 40 μL of SiO_2_ MPs with 160 μL of lipid solution in a small glass vial. The gradient was then induced by the injection of DPBS into the vial at a manual pipetting rate (∼3 mL min^–1^) or at controlled addition rates (600 μL min^–1^ or 60 μL min^–1^) using a syringe pump (KD Scientific). The buffer was added under magnetic stirring at 180 rpm up to a volume of 2 mL, reaching a final solvent:buffer ratio of 10:90 (v/v). The resulting suspension was then heated to 50 °C for 45 min and left to cool down to room temperature. Particles were washed three times in ultrapure water by centrifuging at 50 *g* for 4 min, and were kept at 4 °C until further use. The conditions used during the studies on solvent, buffer addition rate, solvent:buffer ratio, and lipid concentration are specified in detail in Tables S1–4.

### 2.4 Preparation of Erythrocyte Membrane-like Lipid Formulations

Based on lipidomics studies of the outer leaflet of the erythrocyte membrane,^30^ we prepared four lipid formulations of distinct biomimicry complexity. Fluorescent and nonfluorescent versions of the same lipid formulation were prepared. Fluorescent formulations included DT (DOPC, TR-DHPE), CT (DOPC, cholesterol, TR-DHPE), ST (DOPC, cholesterol, sphingomyelin, TR-DHPE), and IT (DOPC, cholesterol, sphingomyelin, phosphatidylinositol, TR-DHPE). Nonfluorescent formulations (D, C, S, and I) included the same lipids at the same lipid ratios, only without TR-DHPE. Detailed information on lipid ratios can be found in Table S5.

### 2.5 OPSALC of SiO_2_ MPs with Membrane-like Formulations

SiO_2_ MPs were prepared at 10 mg mL^–1^ in isopropanol. Fluorescent lipid formulations DT, CT, ST, and IT were prepared at 0.5 mg mL^–1^ in the same solvent. A concentrated lipid-particle suspension was first made by adding 80 μL of SiO_2_ NPs to 320 μL of lipid solutions in small glass vials. The gradient was then induced with the addition of 3.6 mL of DPBS, after which the suspension was heated to 50 °C for 45 min and left to cool down to room temperature. Particles were washed three times in ultrapure water by centrifuging at 50 *g* for 4 min, and finally redispersed in DPBS for analysis.

### 2.6 OPSALC of SiO_2_ NPs with Membrane-like Formulations

SiO_2_ NPs were prepared at 10 mg mL^–1^ in isopropanol. Nonfluorescent lipid formulations D, C, S, and I were prepared at 1.0 mg mL^–1^ in the same solvent. A concentrated lipid-particle suspension was first made by adding 50 μL of SiO_2_ NPs to 450 μL of lipid solutions in small glass vials. The gradient was then induced with the addition of 4.5 mL of DPBS, after which the suspension was heated to 50 °C for 45 min and left to cool down to room temperature. Particles were washed three times in ultrapure water by centrifuging at 10000 *g* for 10 min at 4 °C and finally redispersed in DPBS for analysis.

### 2.7 OPSALC of PCL MPs with Membrane-like Formulations

PCL MPs were produced through a single emulsion solvent evaporation technique. A solution of 0.3% w/v Mowiol 40–88 (25 mL) was prepared in water, to which a 3% w/v solution of PCL in dichloromethane (1 mL) was added. The two-phase mixture was homogenized using tip ultrasonication for 10 s on ice, with a pulse of 1 on 1 s off and an amplitude of 25% (Fisherbrand Q500 Sonicator). The emulsion was stirred at 600 rpm overnight to allow the dichloromethane to evaporate. The resulting suspension was washed three times in ultrapure water by centrifuging at 1500 *g* for 15 min at 4 °C, removing the supernatant, and redispersing in water using a micropipette (Eppendorf). For OPSALC, PCL particles were resuspended in the appropriate amount of isopropanol to yield a concentration of ∼10 mg mL^–1^. Similar to SiO_2_ MPs, a concentrated lipid-particle suspension was made by adding 80 μL of PCL MPs to 320 μL of fluorescent lipid solution (DT, CT, ST, or IT, 1.0 mg mL^–1^) for coating analysis. As an alternative, nonfluorescent lipid solutions (D, C, S, or I, 1.0 mg mL^–1^) were used for the preparation of hemocompatibility assays. The gradient was then induced with the addition of 3.6 mL DPBS while magnetically stirring at 500 rpm. The suspension was further heated to 50 °C for 45 min and left to cool down to room temperature. Particles were washed three times in ultrapure water by centrifuging at 2500 g for 15 min at 4 °C and finally redispersed in DPBS.

### 2.8 Confocal Laser Scanning Microscopy

Uncoated and OPSALC lipid-coated SiO_2_ MP suspensions (0.2–0.4 mg mL^–1^ in DPBS) were imaged using a Zeiss LSM 880 confocal fluorescence microscope. A 20X air objective with a numeric aperture of 0.8 and a 40X water immersion objective with a numeric aperture of 1.2 were used. Image processing and analysis was performed using Fiji software.^48^ Image acquisition parameters (objective, acquisition speed, laser power, gain, and bit depth) and processing methods were kept constant between all the conditions in comparative studies to allow for a direct comparison. For solvent:buffer ratio studies, ethanol and isopropanol conditions were acquired at different laser powers and were therefore only compared qualitatively. For lipid fluorescence intensity quantification, images were acquired with the 20X objective at zoom 1.0 (n = 3 images per condition). To determine the mean fluorescence intensity of TR-DHPE per particle, an automatic threshold was applied to the red-channel image in order to identify each particle as a region of interest (ROI). The mean fluorescence intensity of each ROI was then measured, merged data from a total of n = 3 images per condition, and plotted as the lipid content per particle, per condition, using Origin software (OriginLab). Z-stack images were acquired with a *z*-interval of 0.46 μm with the 40X objective. Orthogonal views of Z-stacks were produced with Fiji software. Coating efficiency was determined as the percentage of red-fluorescent particles (i.e., lipid-coated) per green-fluorescent particles (i.e., all particles) within a specific area of the sample. Calculations were performed for two sample areas across two replicate batches, in a total of n = 557 particles. Where indicated, images were equally adjusted for brightness (i.e., grayscale range adjustment propagated for all images) for improved visualization.

### 2.9 Epifluorescence Microscopy

PCL MPs were analyzed in an ECHO Revolve epifluorescence microscope (ECHO). Images were acquired at 100% light intensity, with an exposure of 10 ms for transmission and 450 ms for Texas Red channels. Images were equally adjusted for Texas Red intensity and merged with transmission images using Fiji software.^48^

### 2.10 Dynamic Light Scattering (DLS) and Zeta Potential (ZP)

The size distribution and zeta potential of lipid-coated SiO_2_ NPs was determined using a ZetaSizer Lab with 90° scatter angle measurements (Malvern). Three particle batches (n = 3) were produced, and each condition of each batch was measured in 5-fold in diluted DPBS (0.05X), at a particle concentration range of 0.05–0.1 mg mL^–1^.

### 2.11 Cryo-transmission Electron Microscopy (cryo-TEM)

Uncoated SiO_2_ NPs, OPSALC lipid-coated SiO_2_ NPs, and lipid vesicle suspensions (approximately 5 mg mL^–1^ in DPBS) were imaged using a FEI Tecnai T20 transmission electron microscope operating at 200 keV. Suspensions were vitrified with liquid ethane (Vitrobot, FEI) and examined on a Gatan cryo-stage. Images were taken under low-dose conditions using a slow scan CCD camera. For better visualization of the coating nanostructure, images were adjusted for brightness and contrast. Particle and vesicle size were quantified using FIJI software.^48^ Lipid coating states were visually identified (“uncoated”, “partially coated”, “coated”, “coated–lamellar”, or “coated–vesicular”) and quantified for the analysis of coating efficiency.

### 2.12 Fluorescence Recovery after Photobleaching (FRAP)

SiO_2_ MPs coated with fluorescent formulations (0.2–0.4 mg mL^–1^) containing TR-DHPE were subjected to FRAP measurements on a Nikon A1 confocal microscope (Nikon) with a 20X objective (numerical aperture 0.75) in the Texas Red channel. Circular ROIs on isolated particles with a diameter of 1 μm (SiO_2_) or 2 μm (PCL) were bleached for ∼2 s (SiO_2_) or ∼100 ms (PCL) using the 488 and 561 nm LASERs simultaneously (SiO_2_) or the 561 nm LASER only (PCL), at maximum power. Reference ROIs, used for fading correction, were always of equal dimensions as their respective bleach ROIs and situated on a different particle. After bleaching, the bleach and reference ROIs were imaged for up to 5 min to record fluorescence recovery. Recovery curves are plotted as ratios of background corrected intensities of bleached ROI/reference ROI as fading correction in time.

### 2.13 Blood Collection

Human whole blood was collected from healthy volunteers at the Blood Donor Service of the Technical Medical Centre of University Twente and used within 4 h of withdrawal. The research did not fall within the scope of the Dutch Medical Research Involving Human Subjects Act. Informed consent was obtained from all volunteers and the used blood collection procedure was approved by the Medical Research Ethics Committee (METC Twente, reference K11–23). For hemolysis and plasma coagulation assays, blood was collected in Vacutainer tubes (BD) containing anticoagulant trisodium citrate with citric acid and dextrose (ACD-A) and treated in the first hour after withdrawal. For multiplex assays, blood was collected in Greiner tubes without additive and immediately anticoagulated with sodium heparin (LEO) to a final concentration of 1.5 IU ml^–1^.

### 2.14 Hemolysis Assays

Erythrocytes (here abbreviated to RBCs, from Red Blood Cells) were isolated based on a protocol described elsewhere.^49^ Briefly, anticoagulated whole blood was first centrifuged at 300 *g* for 2 min, after which the position of the upper layers of plasma and buffy coat were marked on the tube. Both upper layers were removed, substituted with an equal volume of DPBS, and used to redisperse RBCs. Cells were washed three times with DPBS using the same centrifugation conditions and redispersed in the same volume of DPBS. This suspension was then diluted 50 times in the same buffer.

For hemolysis assays, 950 μL of diluted RBC suspensions were incubated with 50 μL of a 4 mg mL^–1^ suspension of uncoated or coated SiO_2_ NPs, at a total particle concentration of approximately 0.2 mg mL^–1^. For the negative controls, RBCs were incubated with 50 μL of DPBS, and for positive controls, cells were incubated with 50 μL of a Triton-X solution. Particle suspensions at the same concentration in pure DPBS were included as controls for absorbance correction. All samples were incubated at 37 °C for 4 h, with gentle mechanical agitation once every hour, and then centrifuged at 300 *g* for 5 min. The supernatant was transferred to a well-plate for quantification of released hemoglobin through absorbance measurements at a wavelength of 540 nm (150 μL, n = 5 measurements per condition). Hemolysis rates (HR) were calculated according to eq 1,
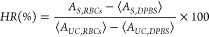
1where A_S,RBCs_ is the absorbance of the supernatant of each condition of SiO_2_ NPs incubated with RBCs; < A_S,DPBS_> is the mean absorbance of the supernatant of the same SiO_2_ NP condition in DPBS; < A_UC, RBCs_> is the mean absorbance of the supernatant of uncoated SiO_2_ NPs incubated with RBCs; and < A_UC,DPBS_> in the mean absorbance of the supernatant of uncoated SiO_2_ NPs in DPBS. The experiment was performed for three different healthy donors.

### 2.15 Plasma Coagulation Time Assay

Platelet-poor plasma (PPP) was isolated from three different healthy donors. After blood withdrawal, blood was centrifuged at 2500 *g* for 15 min. The upper layer of plasma was carefully transferred into new tubes and centrifuged again at 2500 *g* for 15 min. PPP was carefully transferred to Protein LoBind tubes (Eppendorf), aliquoted, and stored at −80 °C. PPP aliquots from three different donors was thawed and pooled on the day of the experiment. For the assay, 450 μL of pooled PPP was incubated with 50 μL of uncoated or coated particle suspensions in DPBS at a total of 0.5 mg per condition. For the negative control, PPP was incubated with 50 μL of DPBS. Samples were incubated for 30 min at 37 °C, after which the plasma-material mixture was carefully mixed 1:1 v/v with recalcification buffer (10 mM HEPES, 20 mM CaCl_2_, 115 mM NaCl, pH 7.2) in a well-plate. Absorbance was measured at 405 nm every minute for 45 min. All tubes and plates were previously blocked with a 0.1 mg mL^–1^ albumin solution prior to contact with plasma.

Plasma coagulation time was determined by fitting the kinetic curves to a Boltzmann model (eq 2) using Origin software,
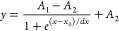
2where A_1_ corresponds to the absorbance baseline prior to coagulation; A_2_ the absorbance plateau after coagulation; and x_0_ the center value, corresponding to the estimated coagulation time, also reported as the “ripening time” in previous reports.^50^

### 2.16 Multiplexed Protein Assay

Whole blood treated with heparin (final concentration 1.5 IU ml-1) was incubated with uncoated and coated PCL MPs, after which platelet-free plasma was isolated and analyzed using a Luminex apparatus. Particle suspensions in DPBS (0.8 mg in 100 μL) were added to 100 μL of DPBS with calcium and magnesium, and 1 mL of whole blood. Samples were incubated for at 37 °C under orbital shaking at 50 rpm in Biopur tubes (Eppendorf) for 2 h. To stop the reaction, a solution of EDTA was added up to a final concentration of 10 mM. Samples were centrifuged at 1500 g for 10 min at room temperature, after which the supernatant was transferred to new tubes and recentrifuged at 13000 g for 10 min. The resulting platelet-free plasma was carefully transferred to cryotubes and stored at −80 °C until analysis.

### 2.17 Statistical Analysis

Normality tests for the analysis of the mean fluorescence intensity of lipids were performed through a Shapiro-Wilk test. Kruskal–Wallis ANOVA with *post hoc* Dunn test, Mann–Whitney test or one-way ANOVA with *post hoc* Tukey tests were performed as indicated in the text. All analyses were performed using Origin software. Statistical significance represented as n.s. (nonsignificant), * (*p* < 0.05), ** (*p* < 0.01), or *** (*p* < 0.001).

## 3 Results and Discussion

### 3.1 Effect of Lipid Concentration on SiO_2_ NP Lipid Coating

To demonstrate the feasibility of the OPSALC method, as well as to allow for its optimization, SiO_2_ NPs were first used. These nanoparticles were selected as the standard substrate due to their surface chemistry, low polydispersity, and small size, which make them an excellent platform for particle colloidal stability studies and characterization of the lipid coating.

**Figure 2:**
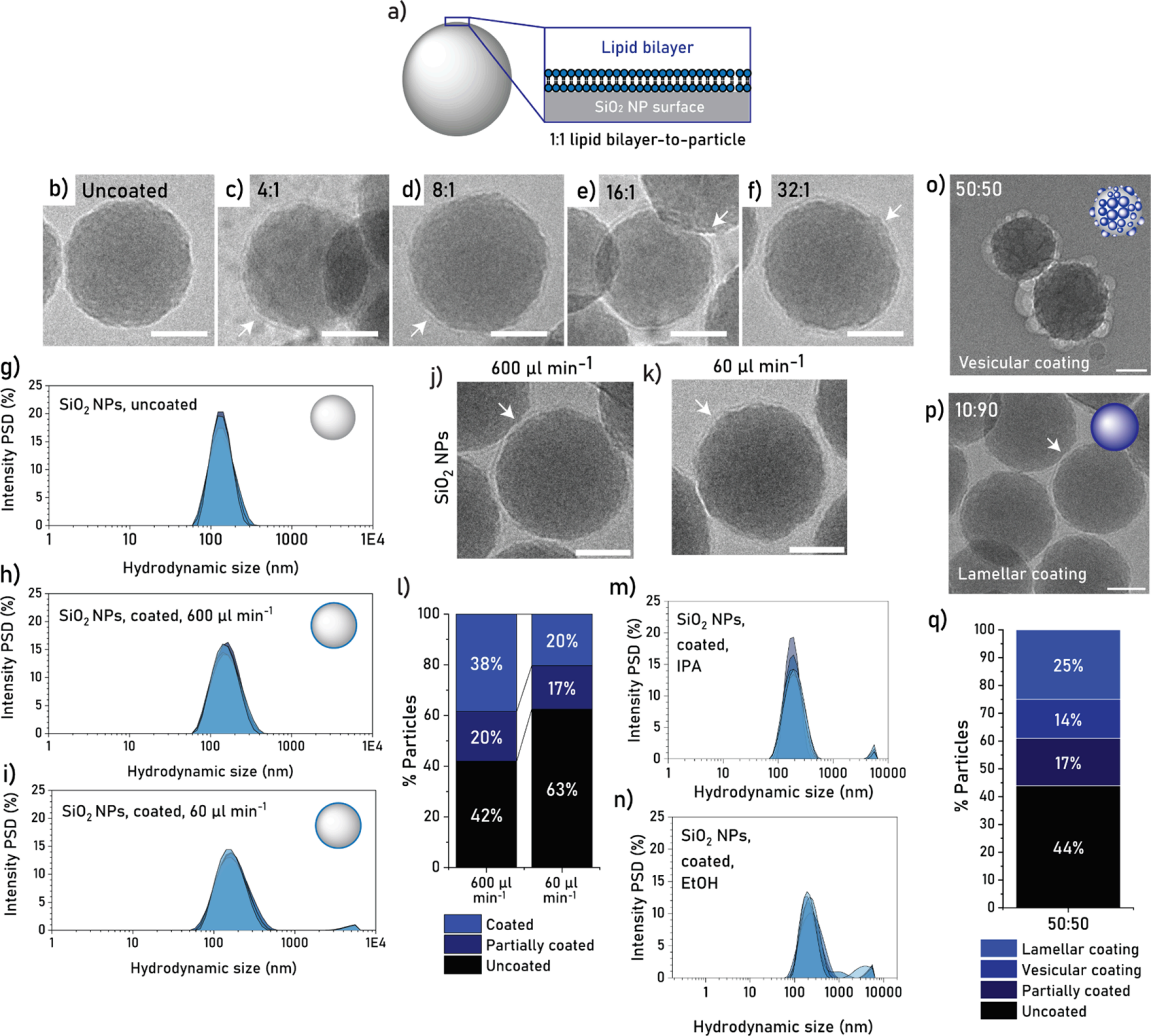
(a) Schematic depiction of a lipid bilayer coating on a SiO_2_ NP. (b–f) Cryo-TEM micrographs of uncoated and coated SiO_2_ NPs with lipid bilayer:particle ratios of 4:1, 8:1, 16:1, and 32:1. An ethanol:DPBS solvent-buffer gradient was used, with a buffer addition rate of 600 μL min^–1^. Arrows indicate lipid layers. Scale bars equal 50 nm. (g–i) Hydrodynamic particle size distribution (PSD) based on intensity of uncoated SiO_2_ NPs (g) and lipid-coated SiO_2_ NPs derived from an ethanol:DPBS gradient formed at buffer addition rates of 600 μL min^–1^ (h) or 60 μL min^–1^ (i). All samples were analyzed prior to washing. Each graph includes five replicate measurements for one representative batch. (j and k) Cryo-TEM micrographs of coated SiO_2_ NPs at 600 and 60 μL min^–1^, respectively, derived from an ethanol:DPBS gradient. Arrows indicate the lipid layer. Scale bars equal 50 nm. (l) Coating efficiency analysis for SiO_2_ NPs coated at 600 and 60 μL min^–1^ buffer addition rates. Calculations based on cryo-TEM images of *n* = 112 and *n* = 128 particles, respectively. Solvent exchange performed at a solvent:buffer ratio of 10:90 (v/v). (m and n) Intensity particle size distribution (PSD) of lipid-coated SiO_2_ NPs produced from isopropanol (IPA) and ethanol (EtOH) gradients, respectively, using a buffer addition rate of 600 μL min^–1^. Samples were analyzed after washing. Each graph includes five replicate measurements for one representative batch. (o and p) Cryo-TEM micrographs of lipid coatings at ethanol:DPBS ratios of 50:50 and 10:90 (v/v), respectively, obtained using a buffer addition rate of >3 mL min^–1^. Arrow indicates a single-layer coating. Scale bars equal 50 nm. (q) Coating type and efficiency analysis for the 50:50 condition, based on *n* = 164 particles.

We studied the effect of lipid concentration on coating formation using lipid solutions of 0.5, 1.0, 2.0, and 4.0 mg mL^–1^, performing dynamic light scattering (DLS) measurements to assess colloidal stability. These lipid concentrations correspond to a mathematical estimation of the number of concentrically stacked lipid bilayers around a single particle, also referred to as lipid bilayer:particle ratios, of 4:1, 8:1, 16:1, and 32:1 (Figure 2a, Methods S1).

Uncoated particles showed a main size intensity peak around 158 nm in DLS studies, whereas SiO_2_ NPs after coating showed main peaks in the range of 161–216 nm (Figure S2, Table S6). These size shifts could indicate the formation of a lipid coating, but also the formation of aggregates. Among coated samples, the increase in lipid concentration from lipid bilayer:particle ratios of 4:1 to 8:1 significantly improved colloidal stability, as observed from the lower polydispersity index (PDI) of the particle suspensions in different batches (Figure S3, Tables S6,7). A further increase in lipid concentration (16:1 and 32:1) did not produce a further improvement in all batches. To determine coating efficiency, cryogenic transmission electron microscopy (cryo-TEM) images were recorded and analyzed. Conditions 8:1 and 32:1 produced the highest percentage of fully coated particles (32% and 31%, respectively, Figure S4). For all fully coated particles, cryo-TEM data revealed a single uniform lipid layer on the particles (Figure 2b–f), confirming that the coating process did not produce multilayers. The observation of single layers also corroborates previous conclusions on solvent exchange methods for planar surfaces.^43,51^ Overall, the most consistent condition in terms of low polydispersity and high coating efficiency was 8:1, with the lowest PDI (mean of 0.146), lowest Z-average (mean of 180.1 nm), and highest coating efficiency (Tables S6,7, Figures S2–4). This condition (8:1), corresponding to 1.0 mg mL^–1^ of lipid solution, was used in all further SiO_2_ NP experiments.

### 3.2 Effect of Buffer Addition Rate on SiO_2_ NP Lipid Coating

Next, we assessed the effect of buffer addition rate on coating formation. We compared the particle size distributions obtained from an addition rate of 600 μL min^–1^ or a rate of 60 μL min^–1^, and observed that the lower rate produced a marginally broader SiO_2_ NP size distribution with some large aggregates (Figure 2g–i). To better understand how lipids assemble with the different buffer addition rates, we also analyzed the lipid vesicles formed in both conditions, in the absence of particles. Similar to the coated particle size distributions, lipid vesicles produced at 60 μL min^–1^ revealed a broader size distribution, whereas 600 μL min^–1^ produced more monodisperse populations (Figure S5a,b, Table S8).

To gain a better understanding of these results, we imaged the coated particles and lipid vesicles using cryo-TEM. Both buffer addition rates produced single-layer lipid coatings (Figure 2j,k); however, the coating efficiency was higher at 600 μL min^–1^ as compared to 60 μL min^–1^ (38% vs. 20% fully coated particles, Figure 2l). In line with the DLS observations, we also confirmed that lipid vesicles produced at 600 μL min^–1^ were smaller and less broad in size distribution than those produced at 60 μL min^–1^, with main size peaks at ∼40 nm and ∼60 nm, respectively (Figure S5c–e). Here, it is worth to note that DLS sizes tend to be larger than cryo-TEM sizes due to the larger hydrodynamic radius observed in DLS and the presence of clusters in suspension; therefore, cryo-TEM data was preferred for vesicle size analysis. The faster addition of water is thought to have induced a faster reduction of the critical micelle concentration of lipids, possibly preventing the system from fully reaching a state of thermodynamic equilibrium. This effect could have likely led to a larger fraction of lipid monomers and smaller lipid assemblies, such as micelles, as compared to larger lipid vesicles, thereby favoring the pathway of lipid-layer deposition during the process (Figure 1a(i)). This effect would also explain the smaller size of lipid vesicles observed at the faster addition rate. This is in agreement with earlier reports that indicate that lipid layers are predominantly formed from adsorbed monomers and micelles.^43,44,47^ Overall, this hypothesis could explain the higher coating efficiency observed at 600 μL min^–1^, and therefore we used this buffer addition rate in all further experiments.

### 3.3 Effect of Solvent on SiO_2_ NP Lipid Coating

Next, we explored the effect of solvent on the formation of a lipid coating by comparing ethanol and IPA buffer gradients. Ethanol was used as solvent in the previous sections, as it represented a good solvent for dispersing SiO_2_ NPs with no aggregation. However, different solvents are known to change the fractions of monomers and lipid assemblies upon buffer addition, influencing the efficiency of the coating process. Based on the differences in dielectric constants for IPA and ethanol, DOPC is expected to result in a larger fraction of lipid monomers and small lipid assemblies in IPA,^52^ boosting the formation of lipid bilayers, as shown in previous studies on planar surfaces.^44^

DLS measurements showed that SiO_2_ NPs were more stable in ethanol than IPA buffer gradients (Figure S6a,b). However, lipid-coated SiO_2_ NPs tended to have a smaller average diameter in IPA than in ethanol (201 nm vs 223 nm, Table S9) as well as a lower occurrence of aggregates (Figure 2m,n). Lipid-coated particles were also analyzed using cryo-TEM, revealing the formation of single-layer coatings on the fully coated particles (Figure S6e, Figure 2d). A more in-depth analysis revealed that the fraction of fully coated SiO_2_ NPs was lower for IPA (18%, *vide infra*Figure 4n) than for ethanol (38%, Figure 2l). In contrast, partially coated SiO_2_ NPs were more frequent in IPA (26%) than in ethanol (20%). The complete formation of a lipid layer in IPA gradients appeared to be slowed down, despite the higher available fraction of lipid monomers and small assemblies. This effect contradicts earlier observations for IPA exchange coatings on planar surfaces,^44^ suggesting that other effects (e.g., lipid–surface, lipid–solvent interactions) may have played a role in coating formation. On the other hand, DLS analysis of lipid vesicles revealed slightly smaller vesicles for IPA (136 nm) than for ethanol (149 nm, Figure S6c,d,f,g, Table S9). This difference in vesicle size is much less pronounced for the different solvents than observed for different buffer addition rates, where vesicle diameters were 97 nm for 600 μL min^–1^ and 212 nm for 60 μL min^–1^ (Table S8). This correlates well with the smaller differences in the quality of coated SiO_2_ NPs comparing IPA and ethanol, while a change in buffer addition rate from 60 μL min^–1^ to 600 μL min^–1^ has a much larger effect on the quality of the coating process.

### 3.4 Effect of Solvent:Buffer Ratio on SiO_2_ NP Lipid Coating

Subsequently, the effect of solvent:buffer ratio on the lipid-coating of SiO_2_ NPs was studied. We imaged SiO_2_ NPs using cryo-TEM after halting the buffer addition process at an ethanol:buffer ratio of 50:50 and 10:90 (Figure 2o,p). For 50:50, 14% of SiO_2_ NPs presented a vesicular coating and 25% presented a full lamellar (i.e., single-layer) coating (Figure 2q), whereas for 10:90 no vesicular coating was observed and 38% of SiO_2_ NPs presented a full lamellar coating (Figure 2l). These cryo-TEM results show that the vesicles in the bulk are attracted to the surface of SiO_2_ NPs to some extent, suggesting a small contribution of vesicle rupture to the formation of lamellar coatings (Figure 1a(ii)). However, at a final solvent to buffer ratio of 10:90, no absorbed vesicles could be observed, which implies that vesicles play a minor role in the coating process. Furthermore, halting the buffer addition at 50:50 will allow the system time to reach equilibrium, therefore allowing a larger fraction of lipids to assemble into micelles and eventually vesicles, as compared to the uninterrupted buffer addition up to 10:90. Taking into account the earlier solvent-exchange studies on planar surfaces,^43,46^ reporting minor to no contribution of vesicle rupture to the coating formation during buffer exchange, vesicles present in the 50:50 case could have also adsorbed to the uncoated parts of a particle surface without necessarily participating in coating formation. These hypotheses, however, warrant a more in-depth biophysical study to unveil the exact contribution of vesicles to coating formation.

Lastly, it is important to address the significant fraction of uncoated (∼40%) and partially coated (∼20%) SiO_2_ NPs observed in cryo-TEM. This phenomenon represents a common issue in the lipid-coating of nanoparticles, and has been similarly reported elsewhere using vesicle fusion techniques.^53,54^ It is our understanding that the natural process of lipid–surface interactions occur at the micelle/vesicle state, and that the natural curvature of nanoparticles reduces the extent of micelle/vesicle adsorption, thus limiting the occurrence of vesicle fusion and lipid bilayer formation. In contrast with other methods, however, the coating efficiency of OPSALC has potential for significant improvement. For example, the use of different lipids could potentially improve lipid–surface attraction and may facilitate lipid packing within the layer. In addition, the use of considerably higher lipid excess to particle surface area should improve the fraction of fully coated particles, as we used 5 × 10^–17^ mg mL^–1^ nm^–2^ while earlier studies on solvent assisted lipid coating of planar surfaces have used 1 × 10^–15^ mg mL^–1^ nm^–2^.^43^

### 3.5 Applying OPSALC to Coat SiO_2_ Microparticles

**Figure 3:**
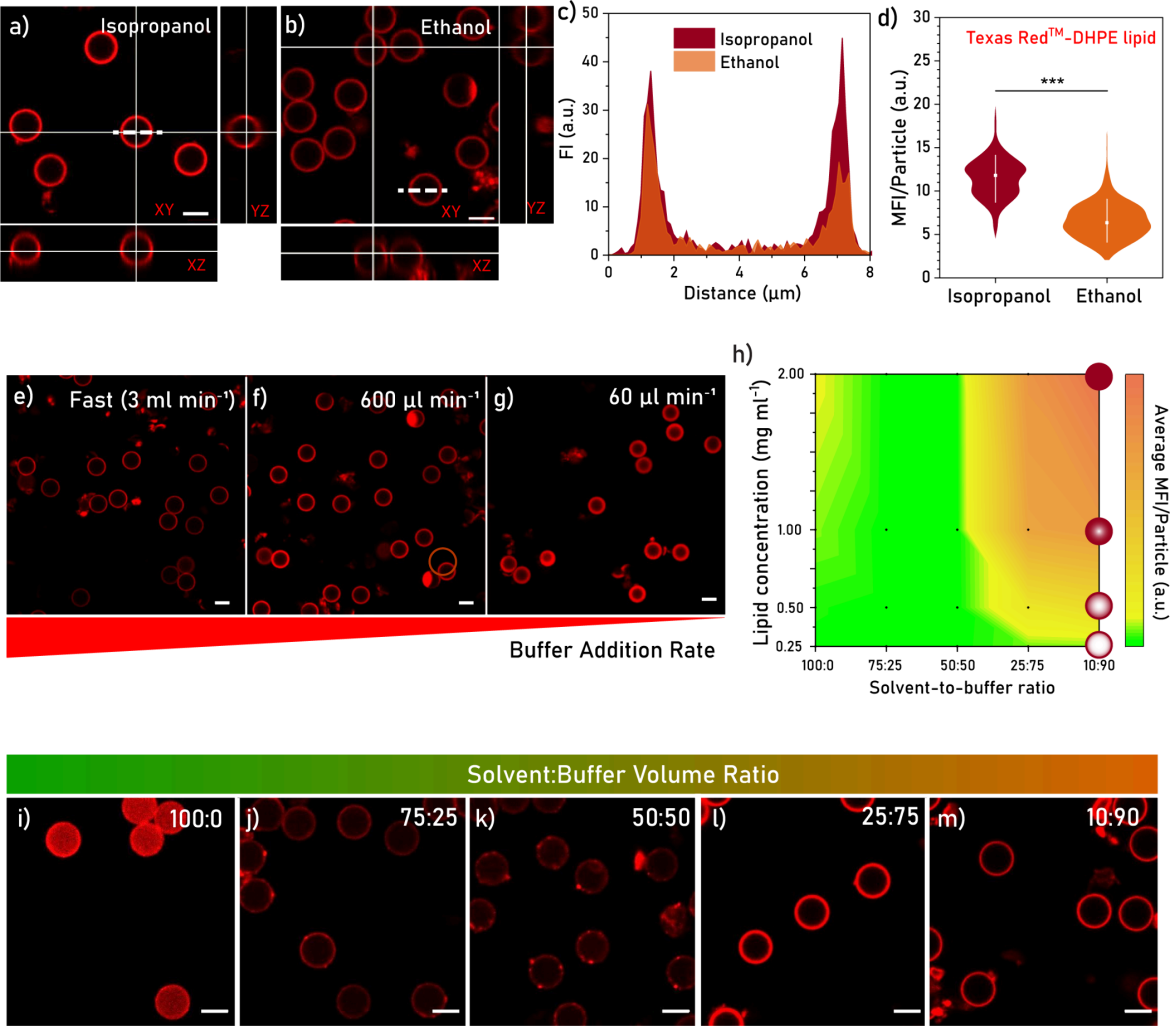
(a and b) Confocal fluorescence micrographs in orthogonal view of lipid coatings generated in IPA and ethanol buffer gradients, respectively. Coatings derived from 0.25 mg mL^–1^ lipid solutions (DOPC with 0.1 mol % TR-DHPE) and a buffer addition rate of 600 μL min^–1^. Images acquired at equal imaging parameters. Dashed lines indicate the plot profile of panel c. Scale bars equal 5 μm. (c) Lipid fluorescence intensity (FI) plot profiles of representative particles coated in IPA or EtOH buffer gradients. (d) Lipid mean fluorescence intensity (MFI) per particle in batches coated in IPA or EtOH buffer gradients. Median dots and 10–90 percentile lines represented in white for *n* = 202 (EtOH) and *n* = 142 (IPA). Statistical analysis performed through a Mann–Whitney test. (e–g) Confocal fluorescence micrographs of lipid coatings generated in fast, 600 μL min^–1^, and 60 μL min^–1^ buffer addition rates, respectively, for an ethanol:DPBS gradient using a 0.25 mg mL^–1^ lipid solution (DOPC with 0.1 mol % TR-DHPE). Scale bars equal 5 μm. (h) Plot of the lipid concentration vs IPA:DPBS ratio of lipid MFI per particle. Black dots represent conditions tested. Cartoons of particles represent different degrees of lipid penetration into the particles. (i–m) Confocal fluorescence micrographs of lipid coatings generated over ethanol:DPBS ratios of 100:0, 75:25, 50:50, 25:75, and 10:90, respectively. Coatings derived from a 0.25 mg mL^–1^ lipid solution (DOPC with 0.1 mol % TR-DHPE) and a buffer addition rate of >3 mL min^–1^. Images l and m have comparable grayscales and were acquired at a lower laser power than images i–k, which have comparable grayscales among themselves, due to the markedly higher coating fluorescence intensity of images l and m. Scale bars equal 5 μm.

Subsequently, we attempted to lipid-coat SiO_2_ MPs using the OPSALC procedure. Similar to coating the nanoparticles, we found that the type of solvent, the rate of addition of buffer, solvent:buffer ratio and lipid concentration were determining our success of achieving a homogeneous lipid coverage on SiO_2_ MPs (Figure 3). Confocal microscopy analysis revealed that the coating efficiency for SiO_2_ MPs was 99.7% (Figure S7). A higher lipid fluorescence per particle was observed for IPA than for ethanol gradients (Figure 3a–d), indicating coatings with a higher lipid content in the case of IPA. This higher lipid fluorescence could indicate a denser or thicker coating, or a better surface coverage, but could also be rationalized with more lipids penetrating the pores of the particles. Homogeneous coatings were found for all tested buffer addition rates using ethanol:buffer gradients (>3 mL min^–1^, 600 μL min^–1^, 60 μL min^–1^, Figure 3e–g). The analysis of fluorescence intensity plot profiles of coated particles revealed different degrees of lipid penetration into the pores of the particles depending on the addition rate, with the lowest condition (60 μL min^–1^) yielding the highest degree of penetration (Figure S8). Similarly, upon increasing lipid concentration, an increase of lipid penetration into the particles was observed (Figure S11). Concentrations of 0.25, 0.5, 1.0, and 2.0 mg mL^–1^ were used, which corresponded to 0.1, 0.2, 0.4, and 0.8 mg per mg of SiO_2_ MPs and n ≈ 44, 88, 176, and 353 lipid bilayers per particle, respectively (**Method S2**). Furthermore, the amount and homogeneity of the coating were substantially different when halting the buffer addition at solvent:buffer ratios of 100:0, 75:25, 50:50, 25:75 and 10:90 for both ethanol and IPA. In the case of ethanol, high intensity (i.e., high lipid content) and homogeneous coatings were observed at buffer contents of ≥75%, whereas at lower buffer contents (<75%) coatings were less homogeneous, showing few high intensity (i.e., high lipid content) features distributed throughout a homogeneous low intensity (low lipid content) coating. (Figure 3i–m, Figures S9,10).

It should be noted that lipid penetration increased, not only with a decrease in buffer addition rate and higher lipid concentration, but also with the use of IPA instead of ethanol, an effect clearly visible for 25:75 solvent:buffer ratios (Figures S10,11, Figure 3h, red circles). Lower buffer addition rates are believed to provide more time for lipid monomers and small lipid assemblies to diffuse into the pores of the particles, whereas higher lipid concentrations and the use of IPA supply higher amounts of lipid monomers and smaller assemblies, which can penetrate the 8–10 nm sized pores. Therefore, we tentatively assign the increased lipid penetration to (i) the availability of higher fractions of lipid monomers and small lipid assemblies, and (ii) the longer time allowed for lipids to diffuse into the pores of the particles.

Altogether, we concluded that the optimal conditions for OPSALC to yield a homogeneous coating on SiO_2_ MPs (with limited penetration) are a moderate excess of lipids (n ≈ 44 lipid bilayers per particle), a minimum buffer addition rate of 600 μL min^–1^, and a final solvent:buffer ratio of 10:90 using either ethanol or IPA as the solvent.

### 3.6 Erythrocyte Membrane-like Lipid Coatings on SiO_2_ MPs

**Figure 4:**
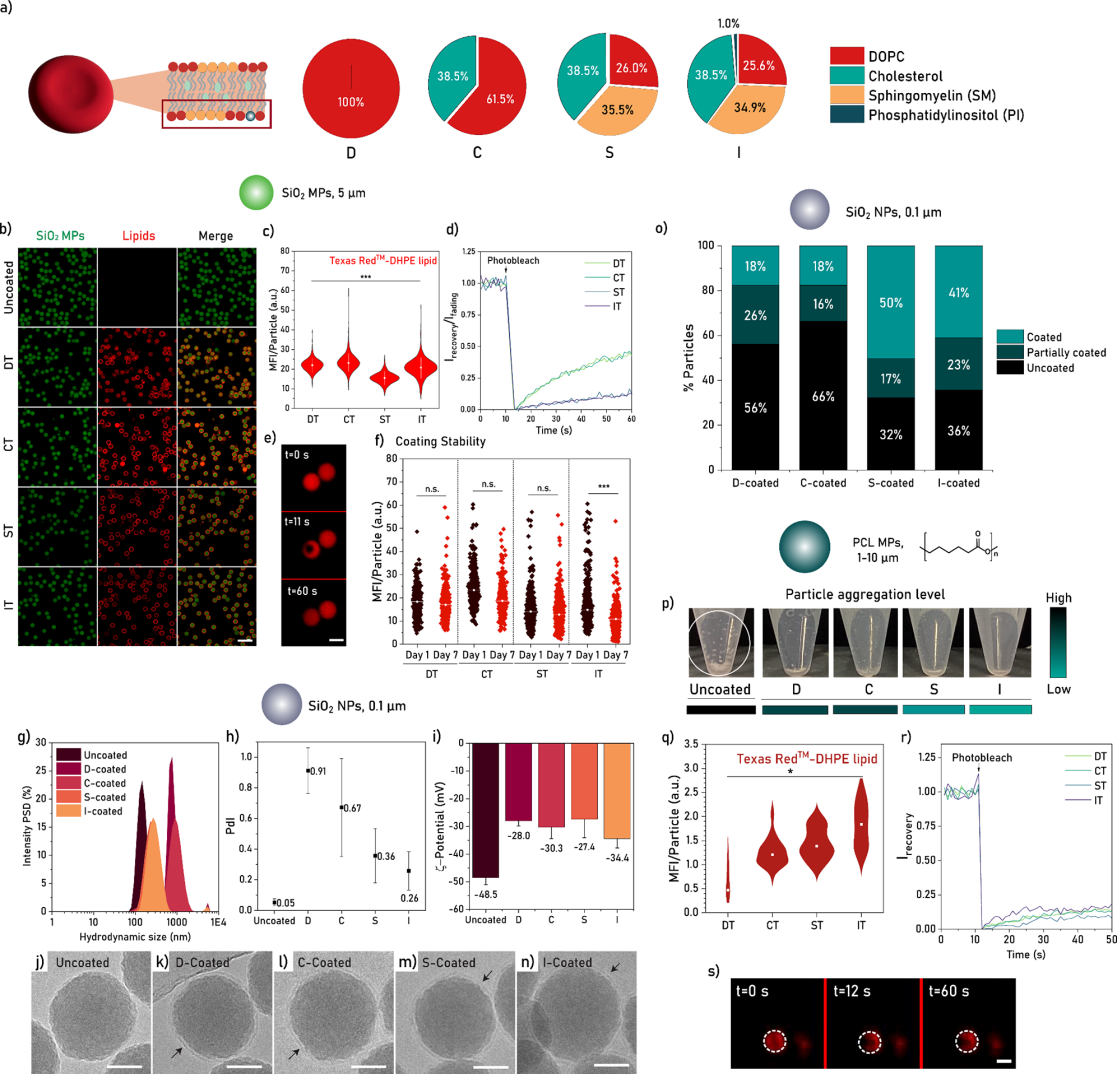
(a) Schematic depiction of the four erythrocyte membrane-like lipid formulations and their respective compositions. (b) Confocal fluorescence micrographs of lipid-coated SiO_2_ MPs using an IPA:DPBS gradient, a buffer addition rate of 600 μL min^–1^, and a lipid concentration of 0.5 mg mL^–1^. The scale bar equals 20 μm. Images equally enhanced for brightness at comparable grayscale ranges. (c) Lipid mean fluorescence intensity (MFI) per particle for each lipid formulation. Median and 10–90 percentile represented in white for *n* = 1633 (DT), 1651 (CT), 2172 (ST), and 2392 (IT) particles. Statistical analysis based on Kruskal–Wallis ANOVA. (d) FRAP traces after bleaching 2 μm diameter spots on lipid-coated SiO_2_ MPs. (e) Confocal fluorescence micrographs of FRAP on a DT-coated SiO_2_ MP before (*t* = 0 s), immediately after (*t* = 11 s), and 60 s after (*t* = 60 s) photobleaching. Images of the Texas Red channel were represented without the green channel to better visualize the effect of FRAP. The scale bar equals 5 μm. (f) Coating stability based on lipid MFI per particle, per formulation, on days 1 and 7. Median per formulation represented in white for *n* = 1350 and 1275 (DT), *n* = 1329 and 969 (CT), *n* = 1872 and 1911 (ST), and *n* = 1911 and 2152 (IT) on days 1 and 7, respectively. (g) Intensity particle size distribution (PSD) of uncoated and coated SiO_2_ NPs using lipid formulations D, C, S, and I. Coatings produced through an IPA:DPBS gradient, using a lipid solution of 1.0 mg mL^–1^ (8:1 lipid bilayer:particle ratio) and a buffer addition rate of 600 μL min^–1^, up to a final solvent:buffer ratio of 10:90 (v/v). (h) Polydispersity index (PDI) of uncoated and coated SiO_2_ NPs. (i) ZP measurements of uncoated and coated SiO_2_ NPs. Data represented as the average of five measurements for *n* = 3 particle batches. (j–n) Cryo-TEM images of uncoated and coated SiO_2_ NPs. The single-layer lipid coating is indicated with an arrow. Images enhanced for brightness with comparable grayscales. Scale bars equal 50 nm. (o) Particle coating efficiency analysis for coatings D, C, S, and I based on cryo-TEM images of *n* = 137, 125, 263, and 224 particles, respectively. (p) PCL MP pellets, uncoated and coated with formulations DT, CT, ST, and IT, redispersed in an aqueous suspension and immediately photographed. (q) Texas Red-lipid mean fluorescence intensity (MFI) per PCL MP coated with DT, CT, ST, or IT using an IPA:DPBS gradient with a buffer addition rate of 600 μL min^–1^ and a lipid concentration of 1.0 mg mL^–1^. White dots represent median values for *n* = 9 (DT), *n* = 64 (CT), *n* = 70 (ST), and *n* = 31 (IT) particles. Statistical analysis was performed through Kruskal–Wallis ANOVA. (r) FRAP traces of lipid-coated PCL MPs. (s) Confocal fluorescence micrographs of IT-coated PCL MPs at *t* = 0 s (before), *t* = 12 s (immediately after), and *t* = 60 s (60 s after) photobleaching. The dotted circle indicates the particle outline. The dark area within the dotted circle represents the photobleached area.

Next, we explored the use of OPSALC in the development of erythrocyte membrane-like coatings on SiO_2_ MPs. Lipidomics studies of the erythrocyte membrane revealed that its outer leaflet, the one in direct contact with blood, has a main composition of phosphatidylcholines (42.3 mol %), sphingomyelins (55.3 mol %), phosphatidylserine (1.5 mol %), and phosphatidylinositol (0.9 mol %),^30^ with a varying concentration of cholesterol (30–40 mol %).^29^ Based on these reports, we selected a phosphatidylcholine lipid (DOPC), a sphingomyelin lipid (egg SM, mainly 16:0 fatty acid tails), a phosphatidylinositol lipid (soy PI, mainly 18:1 fatty acid tails), and cholesterol (Chol) as the components of the cell membrane-like formulations. Phosphatidylserine, although present in the outer leaflet of the membrane, is a typical biomarker of cell aging and certain pathologies^55,56^ and was therefore excluded from the cell membrane design. The different lipids were combined into formulations, according to the lipidomics-derived molar ratios: DOPC only (D), DOPC and Chol (C), DOPC, Chol and egg SM (S); and DOPC, Chol, egg SM and soy PI (I) (Figure 4a). For microscopy, fluorescent versions of these formulations (DT, CT, ST, IT, respectively) were prepared by adding 0.1 mol % of TR-DHPE.

As expected, all fluorescent erythrocyte membrane-like formulations yielded homogeneous lipid coatings on SiO_2_ MPs using the OPSALC method (Figure 4b,c), retaining at least 70% of initial fluorescence intensity for 1 week (Figure 4f, Figure S12). Subsequently, measurements of fluorescence recovery after photobleaching (FRAP) clearly distinguished lipid mobility in the different coatings on SiO_2_ MPs (Figure 4d,e). For DT and CT coatings, nearly identical recovery kinetics were found, with higher lipid mobilities than for ST and IT coatings, which showed near identical recovery kinetics among themselves as well. Obviously, FRAP experiments on microparticles, wherein the bleached area constitutes a significant fraction of the total particle surface area, will show incomplete recovery. The differences in mobility observed in FRAP could be explained by differences in lipid phases formed by the different lipid formulations. Miscibility diagrams of DOPC and cholesterol show that D and C coatings are expected to be in a liquid-disordered phase at room temperature.^57^ In contrast, egg SM has a much higher chain melting temperature (38 °C) than DOPC (−17 °C) and causes mixtures of DOPC, Chol and egg SM, like formulation S and I, to be in the two-liquid phase, containing liquid-disordered and liquid-ordered domains at room temperature.^58^ It has been shown that liquid-ordered phases have significantly lower lipid mobility than liquid-disordered ones, corroborating our findings that D and C coatings are more mobile than S and I coatings. Furthermore, it stands to reason that lipid mixtures (i.e, formulations S and I), which result in partially liquid-ordered layers due to their tighter packing and stronger lipid–lipid attraction, are more prone to form larger, more stable lipid assemblies during OPSALC. This would also mean that lower amounts of lipid monomers and small lipid assemblies are present, limiting the possibility of penetration of lipids into the pores of the particles. This observation, in turn, is in agreement with the smaller penetration depth for S and I, as effectively observed in confocal microscopy (Figure 4b,c). The observed differences show the potential of using OPSALC to tailor the penetration depth by varying not only buffer addition rate, lipid concentration and choice of solvent, but also by varying the phase of the lipid layers.

Overall, the OPSALC method was used to successfully coat SiO_2_ MPs with various erythrocyte membrane-mimicking lipid coatings, with distinct characteristics depending on the exact formulation applied.

### 3.7 Erythrocyte Membrane-like Coatings on SiO_2_ NPs

To confirm that erythrocyte membrane-like formulations could also be applied to nanomaterials, we studied the efficiency of OPSALC on SiO_2_ NPs. Particles were coated through IPA:buffer gradients using formulations D, C, S, and I, and subsequently analyzed using DLS and zeta potential (ZP) measurements. Formulations D and C resulted in a larger particle size distribution than S and I (Figure 4g) and the polydispersity decreased linearly going from D, C, S to I (Figure 4h). All coatings produced a reduction in the ZP, from −48 mV in uncoated particles to approximately −30 mV in coated ones (Figure 4i). Among the tested conditions, particles coated with formulation I produced the lowest ZP value (−34.4 mV) and the lowest polydispersity index (0.26), thus displaying the highest colloidal stability for lipid-coated SiO_2_ NPs. To further characterize the coatings, SiO_2_ NPs were analyzed with cryo-TEM. All formulations successfully produced single-layer coatings on the particles (Figure 4j–n), corroborating our previous cryo-TEM results. Coating efficiency was further quantified as percentage of uncoated, partially coated, and coated particles, revealing that formulations S and I generated a considerably higher percentage of fully coated particles than formulations D and C (D: 18%, C: 18%, S: 50%, I: 41% Figure 4o). Previously, we had proposed that lipid–surface and lipid–medium interactions were the main factor determining the efficiency of OPSALC. Interestingly, we have now observed that lipid–lipid interactions also play a major role in coating efficiency, as formulations with tighter lipid packing (S, I) produce a considerably higher percentage of coated particles. Presumably, the differences in packing and hydration of the partially liquid-ordered layers improved lipid–surface interactions, compensating for the expected occurrence of lower amounts of monomers and small lipid assemblies.

Overall, we confirmed that SiO_2_ NPs can be coated with erythrocyte membrane-like formulations using the OPSALC method, and observed that coating SiO_2_ NPs with formulations producing liquid-ordered and -disordered phases (S, I) was more effective than coating them with formulations producing a liquid-disordered phase only (D, C).

### 3.8 Erythrocyte Membrane-like Coatings on PCL MPs

Next, we attempted to coat hydrophobic PCL MPs using the OPSALC method. PCL was chosen as a hydrophobic particle substrate due to its bioresorbable composition and its common use in drug delivery and tissue engineering. It is often preferred over other hydrophobic polymers (e.g., PLA or PLGA) due to its low cost, high availability, and lower rate of biodegradation.^59,60^ Here, the fluorescent erythrocyte membrane-like formulations used for SiO_2_ MPs were also employed for coating visualization studies.

During the coating process, we observed that coated microparticles were easier to redisperse in water than uncoated microparticles, and that S- and I-coated PCL MPs were significantly easier to redisperse than D- and C-coated PCL MPs (Figure 4p). Fluorescence imaging revealed the highest fluorescence intensity for I-coated PCL MPs, with C- and S-coated particles of only slightly lower intensity, and a significantly lower intensity for D-coated PCL MPs (Figure 4q). FRAP measurements revealed slow recovery rates, comparable for all types of coating (Figure 4r,s), indicating that all lipid layers had limited mobility. We propose that this reduced mobility is due to hydrophobic interactions between the fatty acid tails of the lipids and the surface of PCL particles, which may have resulted in a single immobile monolayer, as suggested elsewhere for another planar hydrophobic substrate.^43^ Altogether, these results indicate that OPSALC can be employed to successfully coat hydrophobic PCL MPs with different mixtures of lipids, resulting in layers with low mobility irrespective of lipid composition.

### 3.9 Hemocompatibility Testing of Lipid-Coated Particles

**Figure 5:**
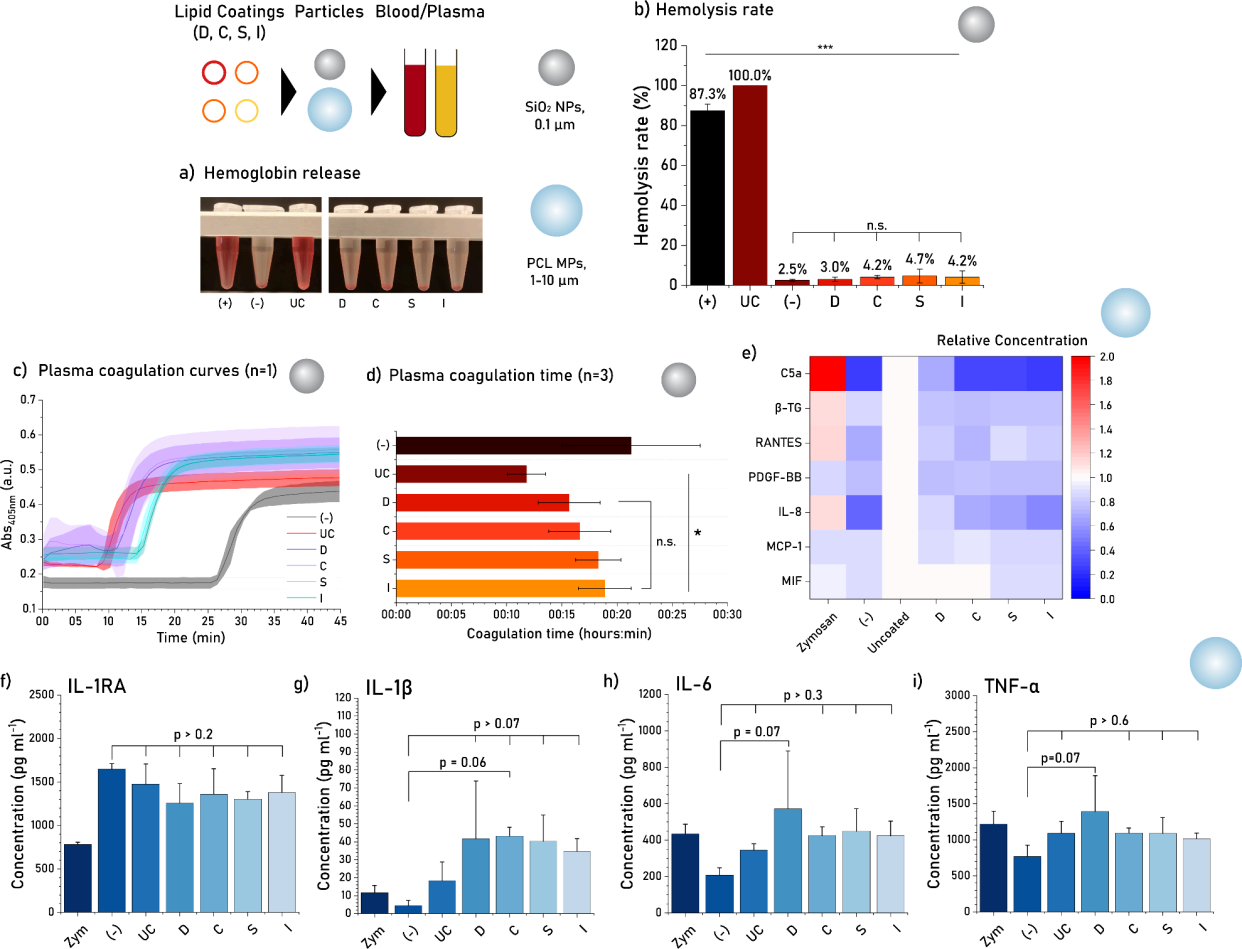
(a) Release of hemoglobin into solution from lysed erythrocytes. Erythrocytes were incubated with Triton-X detergent (+), DPBS buffer (−), uncoated SiO_2_ NPs (UC), or SiO_2_ NPs coated with formulation D, C, S, or I. Coatings produced through an IPA:DPBS gradient, using a lipid solution of 1.0 mg mL^–1^ (8:1 lipid bilayer:particle ratio) and a buffer addition rate of 600 μL min^–1^, up to a final solvent:buffer ratio of 10:90 (v/v). (b) Hemolysis rates determined from the absorbance of released hemoglobin. Data normalized to uncoated SiO_2_ NP-induced hemolysis and represented by the average of 5-fold measurements for *n* = 3 particle batches. Statistical analysis made with one-way ANOVA with a post hoc Tukey test. (c) Plasma coagulation curves from fibrin clot formation kinetics. Plasma incubated with buffer (−), uncoated SiO_2_ NPs (UC), or coated SiO_2_ NPs with formulation D, C, S, or I. Data represented for five replicate measurements. (d) Determined coagulation times from fibrin clot formation kinetics. Data represented by the average of 5-fold measurements for *n* = 3 particle batches. Statistical analysis was performed through one-way ANOVA with a post hoc Tukey test. (e) Normalized heat map of whole blood biomarker analysis after incubation with uncoated PCL MPs (reference, in white) or PCL MPs coated with lipid formulation D, C, S, or I. Biomarkers for complement activation (C5a), platelet activation (β-TG, PDGF-BB), and leukocyte recruitment (IL-8, RANTES, MCP-1, MIF). (f–i) Inflammatory biomarker concentrations (IL-1RA, IL-1β, IL-6, and TNF-α, respectively) after incubation with uncoated (UC) and coated (D, C, S, or I) PCL MPs. Zym: zymosan-stimulated blood, a complement activator. (−): nonstimulated blood. Data represented by the average of 3-fold measurements for *n* = 3 particle batches. Statistical analysis was performed through one-way ANOVA with a post hoc Tukey test.

To demonstrate the functionality of distinct lipid coatings, the effect of lipid formulation on particle hemocompatibility was investigated. We first conducted hemolysis and plasma coagulation assays on SiO_2_ NPs, as they are known for their highly hemolytic and procoagulant nature.^61,62^ Hemolysis assays showed that uncoated SiO_2_ NPs are extremely hemolytic at particle concentrations as low as 0.2 mg mL^–1^, and specifically more hemolytic than the positive control (1% v/v Triton-X final concentration), which led to its use as the reference for hemolysis rate calculation. In contrast, SiO_2_ NPs coated with any of the lipid formulations yielded a drastic reduction of erythrocyte damage, coupled to a reduction in the amount of hemoglobin release and the resulting hemolysis rates (from 100% to below 5%, Figure 5a,b). Moreover, no significant difference was observed between lipid formulations and the negative control, confirming its inert behavior. It must be noted, however, that the particle aggregation observed for D- and C-coated SiO_2_ NPs (Figure 4g) could be partially responsible for the reduction in hemolysis rate. As a result, the low hemolysis rates of coatings S and I are more reliable than the hemolysis rates of coatings D and C, as they more accurately indicate the hemoprotective effect of the lipid layer on single particles.

To determine whether the lipid composition of the particle coatings have an effect on plasma coagulation time, coated and uncoated SiO_2_ NPs were incubated with platelet-poor plasma, recalcified, and analyzed via the increase in scattering during fibrin clot formation. SiO_2_ surfaces are known for their procoagulant properties through the surface-induced activation of factor XII, or Hageman factor, an important mediator of the intrinsic coagulation pathway.^63,64^ Plasma coagulation curves demonstrated that S and I coatings could induce a more pronounced delay in coagulation onset than D and C coatings (Figure 5c), demonstrating a more significant attenuation of the pro-coagulating properties of SiO_2_ NPs, although this advantage was not consistently observed. The coagulation curves for two other tested batches can be found in Figure S13. Plasma coagulation time was then calculated as average of all batches tested. Overall, the average coagulation time induced by different coatings was not significantly different; however, a significant difference was observed between the uncoated and all coated conditions (Figure 5d). Collectively, these results indicate that all tested coatings were effective in attenuating the coagulating effect of SiO_2_ NPs.

To further study the hemocompatibility effects of the lipid coatings, we explored the complement activation, platelet activation, leukocyte recruitment, and inflammation response induced by uncoated vs lipid-coated PCL MPs. Despite the low toxicity of this polymer, hydrophobic surfaces are known to adsorb fibrinogen, IgG, and C3, proteins involved in platelet/leukocyte adhesion and complement activation.^65,66^ Since hydrophobic micromaterials have been researched as drug carriers,^67,68^ porous functional scaffolds,^69^ and, more recently, oxygen-generating components of tissue engineered implants,^70,71^ hemocompatibility has become a key aspect of their biocompatibility.

Indeed, PCL MPs stimulated the production of all complement activation, platelet activation, and leukocyte recruitment markers when compared to nonstimulated blood (Figure 5e). The relatively high levels of C5a, a common biomarker for all complement activation pathways,^65^ indicated that PCL MPs tend to induce complement activation to some extent. Lipid coatings, however, attenuated this complement-activating effect with an interesting trend: increasingly complex lipid formulations produced a gradually safer response. Neutrophil chemotactic marker IL-8^72^ followed a similar trend, with more complex lipid coatings resulting in lower IL-8 production. Platelet activation markers (β-TG and PDGF-BB),^65,73^ platelet-secreted monocyte and lymphocyte attractant RANTES,^74^ and monocyte attractant MCP-1^75^ were found at generally low levels for all coating formulations. Finally, activated T-cell cytokine MIF^76^ was found at similarly high levels in uncoated, D-coated and C-coated PCL MPs, but found at lower levels in S-coated and I-coated PCL MPs. These results suggested that PCL MPs were not entirely bioinert in blood, and that lipid coatings with more complex erythrocyte membrane mimicry were able to notably improve PCL MP hemocompatibility. To expand on these results, the levels of inflammatory biomolecules (IL-1RA, IL-1β, IL-6, and TNF-α) were also analyzed. While there was no statistically significant difference between the negative control and any PCL MPs for any of these biomarkers, there was a consistent tendency for D-coated PCL MPs to have an almost statistical difference from the negative controls (Figure 5f–i), indicating a slightly more pro-inflammatory profile than uncoated and other coated particles.

Ultimately, all results corroborated that S and I lipid formulations generated a better improvement on blood-contacting particle hemocompatibility as compared to D and C lipid formulations, indicating a potential benefit in using biomimetically complex lipid coatings in biomedical applications.

## 4 Conclusions

We demonstrated that on-particle solvent-assisted lipid coating (OPSALC) can be successfully used to lipid-coat particles of different sizes and materials. The efficiency of the OPSALC method depends, among others, on the extent of the solvent:buffer gradient, on the fractions and types of lipid species formed throughout the process, and on the balance of lipid–particle and lipid–medium interactions. Moreover, we demonstrate that OPSALC enables a facile and scalable lipid-coating of particles using biologically relevant lipid formulations in a safer, more sustainable, and ethically more acceptable manner than natural cell membrane coatings. Different erythrocyte membrane-like lipid formulations have been tested, offering new entries into cell membrane engineering when designing blood-contacting lipid coatings. Moreover, we demonstrated that erythrocyte membrane-like lipid formulations can significantly improve particle hemocompatibility, as well as enhance the colloidal stability of hydrophobic microparticles in water. Particles coated with synthetic cell membranes can be incorporated in the design of novel drug delivery systems, engineering cell mimics, building blocks for tissue engineering, and medical devices, providing a hemocompatibility enhancement toolbox for future biomedical and pharmaceutical innovation.

## Supporting Information Available

Additional figures, methods, and tables on the solvent-based lipid coating method (PDF)

## Abbreviations

β-TG: β-thromboglobulin
D: lipid formulation of pure DOPC
DLS: dynamic light scattering
DOPC: 1,2-dioleoyl-*sn*-glycero-3-phosphocholine
DT: fluorescent version of formulation D
DPBS: Dulbecco’s phosphate-buffered saline
C: lipid formulation of DOPC and cholesterol
CT: fluorescent version of formulation C
FI: fluorescence intensity
FRAP: fluorescence recovery after photobleaching
I: lipid formulation of DOPC, cholesterol, sphingomyelin, and phosphatidylinositol
IT: fluorescent version of formulation I
IL-1β: interleukin 1 β
IL-1RA: interleukin 1 receptor antagonist
IL-6: interleukin 6
IL-8: interleukin 8
IPA: isopropanol
MCP-1: monocyte chemoattractant protein-1
MFI: mean fluorescence intensity
MIF: macrophage migration inhibitory factor
MP: microparticle
NP: nanoparticle
n.s.: nonsignificant
PC: phosphatidylcholine
PCL: polycaprolactone
PDGF-BB: platelet-derived growth factor BB subunits
PE: phosphatidylethanolamine
PI: phosphatidylinositol
PLA: polylactic acid
PS: phosphatidylserine
RANTES: regulated upon activation, normal T cell expressed and presumably secreted chemokine
S: lipid formulation of DOPC, cholesterol, and sphingomyelin
SALB: solvent-assisted lipid bilayer method
SM: sphingomyelin
ST: fluorescent version of formulation S
TEM: transmission electron microscopy
TNF-α: tumor necrosis factor α
TR-DHPE: Texas Red 1, 2-dihexadecanoyl-*sn*-glycero-3-phosphoethanolamine
UC: uncoated
ZP: zeta potential
Zym: zymosan
