# Supplementary material for: OPSALC: On-Particle Solvent-Assisted Lipid Coating to Create Erythrocyte Membrane-like Coatings with Improved Hemocompatibility
Source: ACS Appl Mater Interfaces. 2025 Mar 13;17(12):18179–93. doi: 10.1021/acsami.5c02103 (PMC11955951; doi:10.1021/acsami.5c02103)
Supplement: Supplementary file 1 — am5c02103_si_001.pdf [file am5c02103_si_001.pdf]

Supporting Information

OPSALC: On-Particle Solvent-Assisted Lipid Coating to Create Erythrocyte Membrane-Like Coatings with Improved Hemocompatibility

Francisca L. Gomes[1,2], Dorothee Wasserberg[2,3], Rick Edelbroek[2], Jasper van Weerd[3], Pascal Jonkheijm[2*†], and Jeroen Leijten[1*†]

[1] Department of Bioengineering Technologies, Leijten Laboratory, Faculty of Science and Technology, University of Twente, Drienerlolaan 5, Enschede, 7522NB The Netherlands

[2] Department of Molecules and Materials, Laboratory of Biointerface Chemistry, Faculty of Science and Technology, University of Twente, Drienerlolaan 5, Enschede, 7522NB The Netherlands

[3] LipoCoat BV, Hengelosestraat 535, Enschede, 7521AG The Netherlands

[*]Authors contributed equally.

[†]Corresponding authors: p.jonkheijm@utwente.nl; jeroen.leijten@utwente.nl

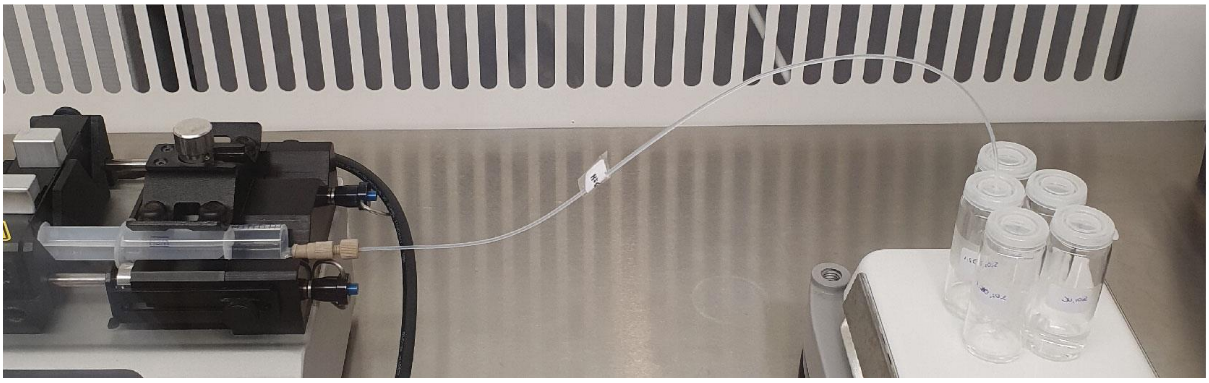

Figure S1. Experimental setup of the on-particle solvent-assisted lipid coating (OPSALC) method. Pump-mediated addition of buffer to a lipid-particle mixture in a water- miscible alcohol.

Table S1. OPSALC conditions for the study of the effect of solvent type on lipid coating formation.

**Gradient study: Solvent type**	**Sample**	**Solvent type**	**Buffer addition rate (µl min^{-1})**	**Solvent-to-buffer (v/v)**	**Lipid concentration (mg ml^{-1})**
	1	Isopropanol	600	10:90	0.25
	2	Ethanol	600	10:90	0.25

Table S2. OPSALC conditions for the study of the effect of buffer addition rate on lipid coating formation.

**Gradient study: Buffer addition rate**	**Sample**	**Solvent type**	**Buffer addition rate (µl min^{-1})**	**Solvent-to-buffer (v/v)**	**Lipid concentration (mg ml^{-1})**
	1	Ethanol	Fast (>3000)	10:90	0.25
	2	Ethanol	600	10:90	0.25
	3	Ethanol	60	10:90	0.25

Table S3. OPSALC conditions for the study of the effect of solvent-to-buffer ratio on lipid coating formation.

**Gradient study: Solvent-to-buffer ratio**	**Sample**	**Solvent type**	**Buffer addition rate (µl min^{-1})**	**Solvent-to-buffer (v/v)**	**Lipid concentration (mg ml^{-1})**
	1	Ethanol	Fast (>3000)	100:0	0.25
	2	Ethanol	Fast	75:25	0.25
	3	Ethanol	Fast	50:50	0.25
	4	Ethanol	Fast	25:75	0.25
	5	Ethanol	Fast	10:90	0.25

Note: study also performed in isopropanol; results are shown in Suppl. Fig. 4.

Table S4. OPSALC conditions for the study of the effect of lipid concentration on lipid coating formation.

	**Sample**	**Solvent type**	**Buffer addition rate (µl min^{-1})**	**Solvent-to-buffer (v/v)**	**Lipid concentration (mg ml^{-1})**
**Gradient study: Lipid concentration**	1	Isopropanol	Fast (>3000)	100:0	0.25
	2	Isopropanol	Fast	100:0	0.5
	3	Isopropanol	Fast	100:0	1.0
	4	Isopropanol	Fast	100:0	2.0
	5	Isopropanol	Fast	75:25	0.25
	6	Isopropanol	Fast	75:25	0.5
	7	Isopropanol	Fast	75:25	1.0
	8	Isopropanol	Fast	75:25	2.0
	9	Isopropanol	Fast	50:50	0.25
	10	Isopropanol	Fast	50:50	0.5
	11	Isopropanol	Fast	50:50	1.0
	12	Isopropanol	Fast	50:50	2.0
	13	Isopropanol	Fast	25:75	0.25
	14	Isopropanol	Fast	25:75	0.5
	15	Isopropanol	Fast	25:75	1.0
	16	Isopropanol	Fast	25:75	2.0
	17	Isopropanol	Fast	10:90	0.25
	18	Isopropanol	Fast	10:90	0.5
	19	Isopropanol	Fast	10:90	1.0
	20	Isopropanol	Fast	10:90	2.0

Table S5. Detailed compositions of erythrocyte-mimicking lipid coating formulations. Fluorescent formulations (DT, CT, ST and IT) and non-fluorescent formulations (D, C, S and I) composed by different molar ratios of DOPC, cholesterol, egg sphingomyelin, soy phosphatidylinositol, and Texas Red™-DHPE.

Formulation name	TR-DHPE (mol%)	DOPC (mol%)	Cholesterol (mol%)	Sphingomyelin (mol%)	Phosphatidyl-inositol (mol%)
**DT**	0.1	99.9	-	-	-
**CT**	0.1	61.4	38.5	-	-
**ST**	0.1	25.9	38.5	35.5	-
**IT**	0.1	25.5	38.5	34.9	1.0
**D**	-	100	-	-	-
**C**	-	61.5	38.5	-	-
**S**	-	26.0	38.5	35.5	-
**I**	-	25.6	38.5	34.9	1.0

Table S6. First batch for lipid concentration studies. Dynamic light scattering data of SiO_2 NPs, uncoated and coated with different lipid bilayer-to-particle ratios, after washing. Data represented as mean ± standard deviation of five replicate measurements.

Sample	PdI	Z-average (nm)
**SiO_2 NPs, uncoated**	0.169 ± 0.017	162.2 ± 2.4
**SiO_2 NPs, coated 4:1**	0.324 ± 0.011	235.9 ± 2.4
**SiO_2 NPs, coated 8:1**	0.126 ± 0.026	173.0 ± 1.2
**SiO_2 NPs, coated 16:1**	0.194 ± 0.004	206.0 ± 1.9
**SiO_2 NPs, coated 32:1**	0.126 ± 0.019	193.2 ± 0.8

Table S7. Second batch for lipid concentration studies. Dynamic light scattering data of SiO_2 NPs, uncoated and coated with different lipid bilayer-to-particle ratios, after washing. Data represented as mean ± standard deviation of five replicate measurements.

Sample	PdI	Z-average (nm)
**SiO_2 NPs, uncoated**	0.061 ± 0.008	134.7 ± 1.7
**SiO_2 NPs, coated 4:1**	0.253 ± 0.018	211.1 ± 3.1
**SiO_2 NPs, coated 8:1**	0.166 ± 0.013	187.2 ± 2.8
**SiO_2 NPs, coated 16:1**	0.242 ± 0.020	212.7 ± 1.9
**SiO_2 NPs, coated 32:1**	0.216 ± 0.014	197.6 ± 2.2

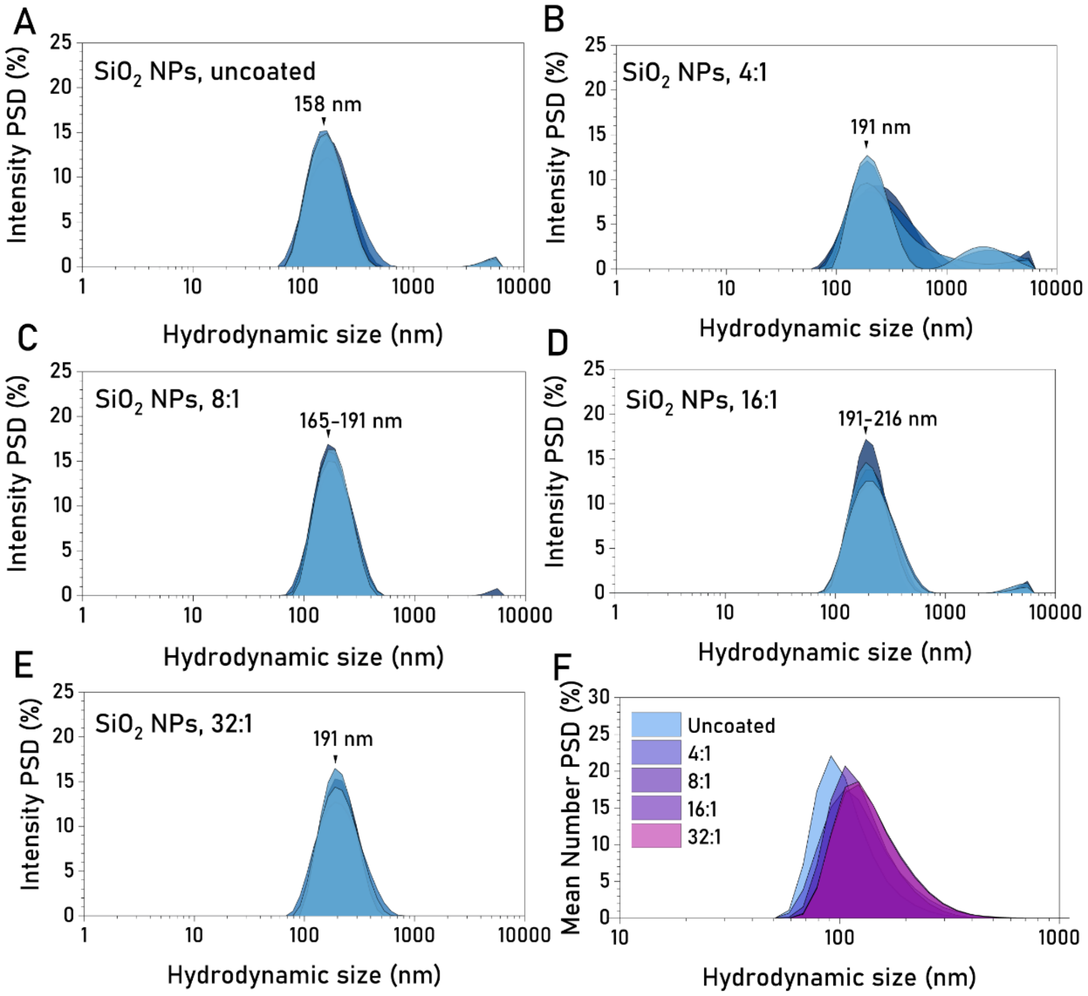

Figure S2. First batch for coating silica particles with lipids using different lipid concentrations. **A-E.** Intensity particle size distribution (PSD) of coated SiO_2 NPs. Curves are five replicate measurements. **F.** Mean number PSD of silica particles coated with lipids using different lipid concentration studies after washing. For each condition, the average of five replicate measurements is shown.

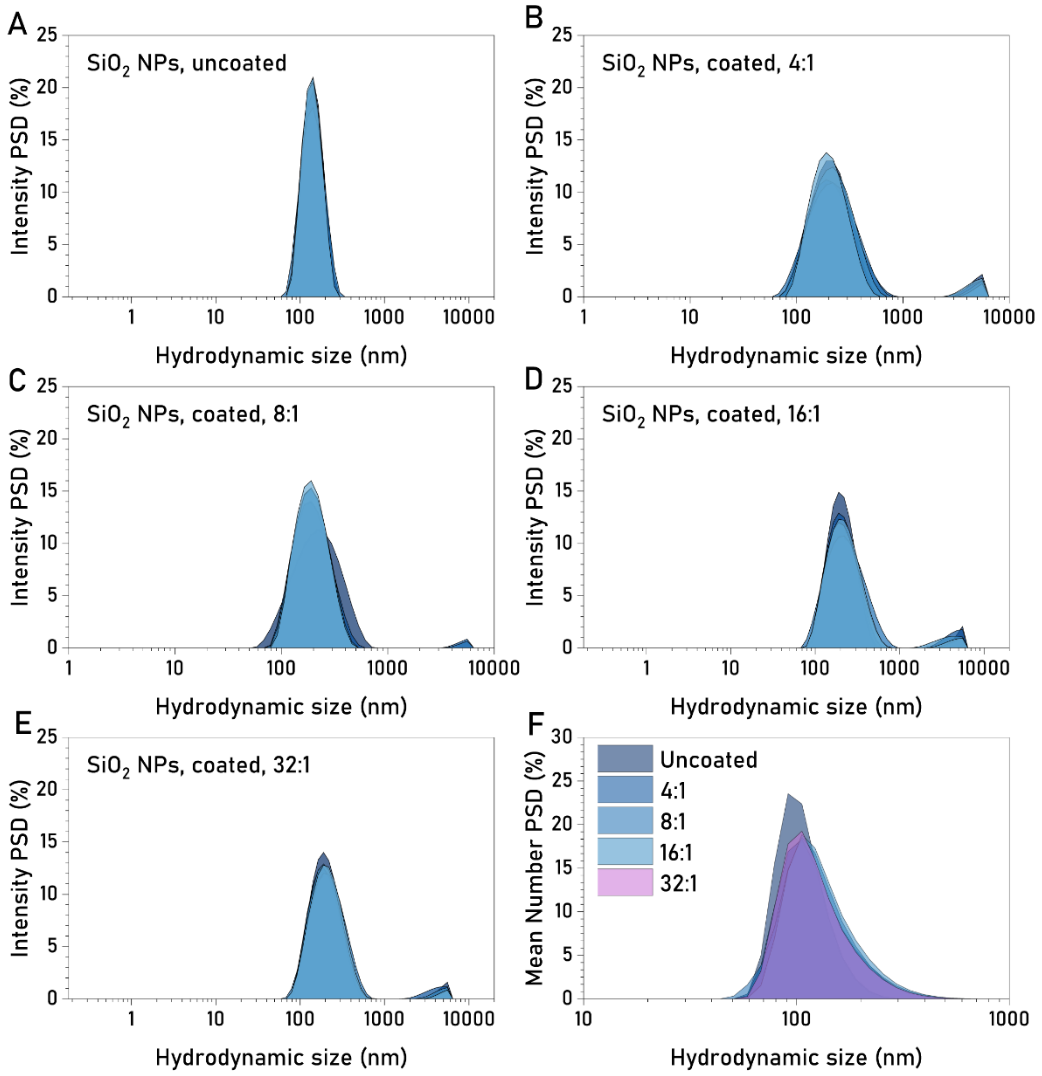

Figure S3. Second batch for coating silica particles with lipids using different lipid concentration studies. **A-E.** Intensity particle size distribution (PSD) of lipid concentration studies after washing. Curves represent five replicate measurements. **F.** Mean number PSD of silica particles coated with lipids using different lipid concentration after washing. Each condition was averaged for five replicate measurements.

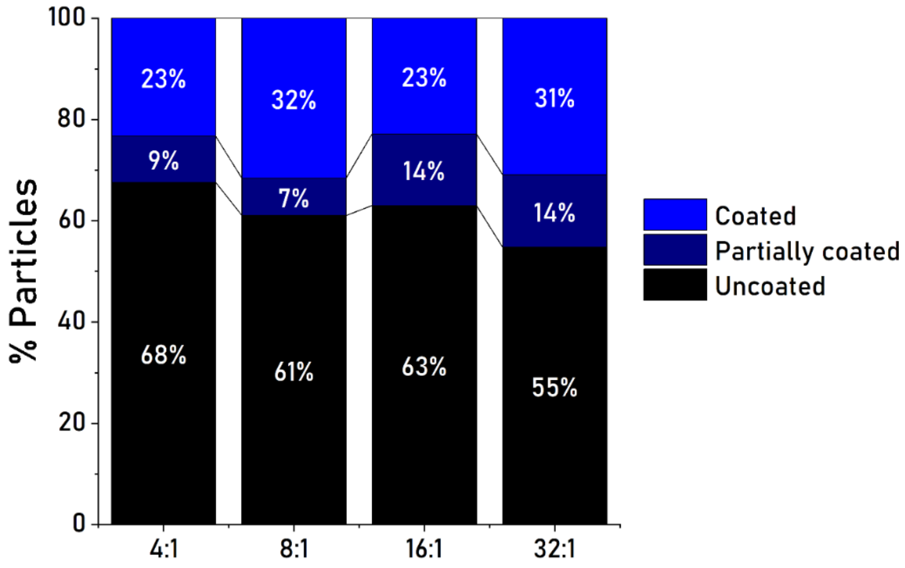

Figure S4. Coating efficiency analysis for different lipid concentrations (0.5, 1.0, 2.0, and 4.0 mg ml^{-1}, corresponding to 4:1, 8:1, 16:1, and 32:1 lipid bilayers per particle, respectively), based on a semi-quantification of cryo-TEM images. Coatings produced from ethanol:DPBS gradients at a buffer addition rate of 600 µl min^{-1}. Data represented for n = 176, 136, 219, and 84 particles for conditions 4:1, 8:1, 16:1, and 32:1, respectively.

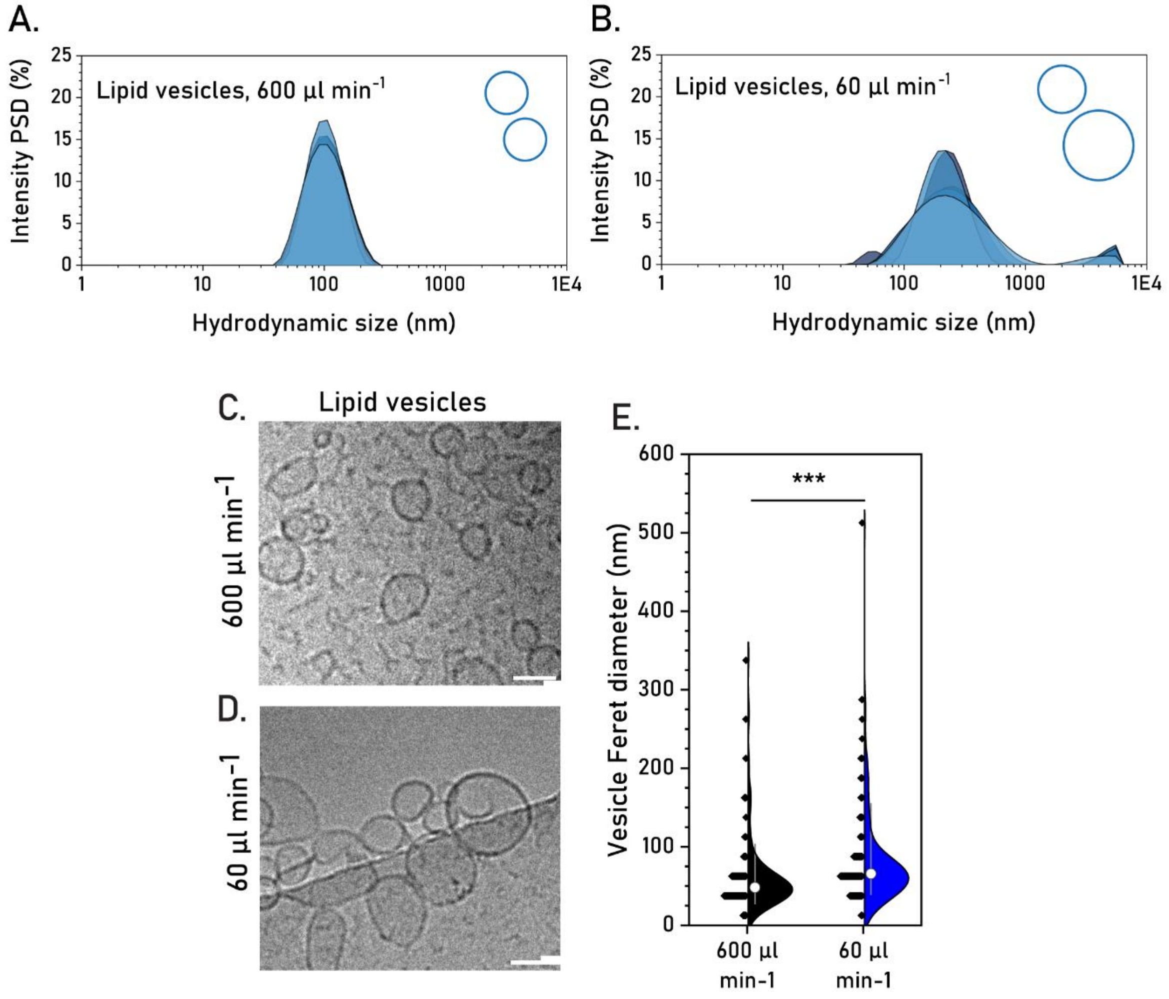

Figure S5. A,B. Intensity particle size distribution (PSD) of lipid vesicles produced from an ethanol:DPBS gradient with buffer addition rates of 600 µl min^{-1} (A) or 60 µl min^{-1} (B). Samples were analyzed prior to washing. Each graph includes five replicate measurements. **C,D.** Cryo-TEM images of lipid vesicles resulting from the two buffer addition rates. **E.** Feret diameter of lipid vesicles resulting from 600 µl min^{-1} and 60 µl min^{-1} addition rates, calculated based on cryo-TEM images of n = 186 and n = 180 vesicles, respectively. White dot represents median, line represents extent of data. Statistical analysis performed through Mann-Whitney test.

Table S8. Dynamic light scattering data of SiO_2 NPs, uncoated and coated with different buffer addition rates (600 µl min^{-1}, 60 µl min^{-1}), prior to washing as well as controls without SiO_2 NPs (lipid vesicles). Coatings obtained from ethanol:DPBS gradients using a lipid concentration of 1.0 mg ml^{-1} (8:1 lipid bilayers to particle). Data represented as mean ± standard deviation for five replicate measurements. Representative batch for buffer addition rate studies.

Sample	PdI	Z-average (nm)
SiO_2 NPs, uncoated	0.078 ± 0.020	128.5 ± 0.7
SiO_2 NPs, coated, 600 µl min^{-1}	0.118 ± 0.020	146.0 ± 2.4
SiO_2 NPs, coated, 60 µl min^{-1}	0.194 ± 0.010	161.3 ± 2.4
Lipid vesicles, 600 µl min^{-1}	0.108 ± 0.025	96.6 ± 1.3
Lipid vesicles, 60 µl min^{-1}	0.316 ± 0.027	211.9 ± 4.1

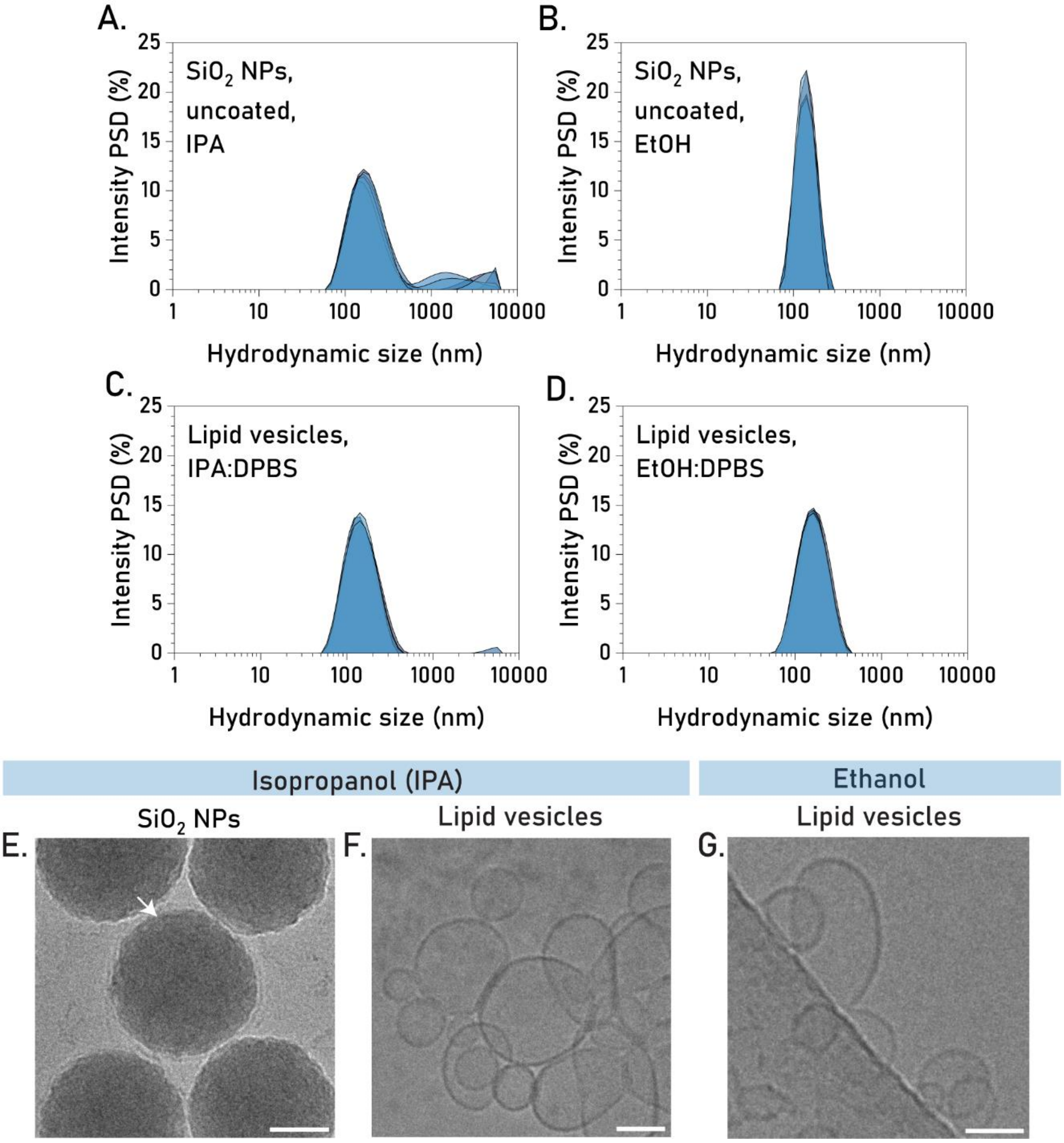

Figure S6. A,B. Intensity particle size distribution (PSD) of uncoated SiO_2 NPs from isopropanol (IPA) or ethanol (EtOH) buffer gradients. Samples were analyzed after washing. Each graph includes five replicate measurements. **C,D**. Intensity PSD of lipid vesicles produced in IPA or EtOH buffer gradients. Samples were analyzed without washing. Each graph includes five replicate measurements. **E**. Cryo-TEM image of lipid-coated SiO_2 NPs. White arrow indicates lipid coating. **F.** Cryo-TEM image of lipid vesicles derived from an IPA buffer

gradient. **G.** Cryo-TEM image of lipid vesicles derived from an ethanol buffer gradient. All scale bars equal 50 nm.

Table S9. Dynamic light scattering data of SiO_2 NPs, uncoated and coated using ethanol (EtOH) or isopropanol (IPA) gradients, after washing. Vesicles analyzed prior to washing. Data represented as mean ± standard deviation for five replicate measurements. Representative batch for solvent studies.

Sample	PdI	Z-average (nm)
SiO_2 NPs, uncoated, IPA	0.303 ± 0.014	182.5 ± 2.3
SiO_2 NPs, uncoated, EtOH	0.047 ± 0.019	135.0 ± 2.4
SiO_2 NPs, coated, IPA	0.276 ± 0.034	201.2 ± 4.9
SiO_2 NPs, coated, EtOH	0.277 ± 0.030	223.2 ± 3.9
Lipid vesicles, IPA	0.142 ± 0.018	135.5 ± 1.7
Lipid vesicles, EtOH	0.126 ± 0.006	148.5 ± 2.6

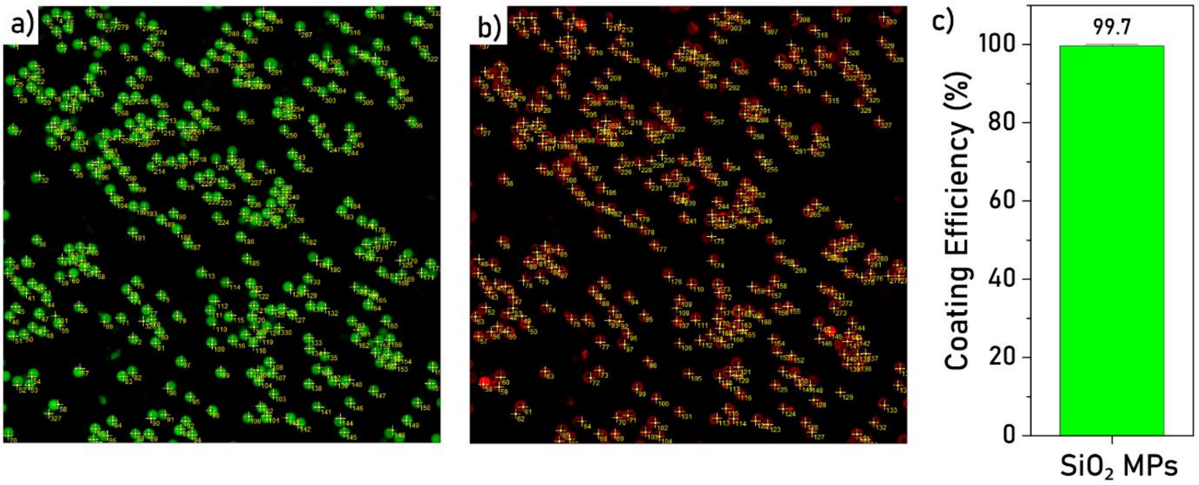

Figure S7. a,b. Green and red channel particle counting, respectively, to determine the percentage of coated SiO_2 MPs. **c.** Percentage of coated SiO_2 MPs. Data acquired for two replicate batches, with n = 557 particles.

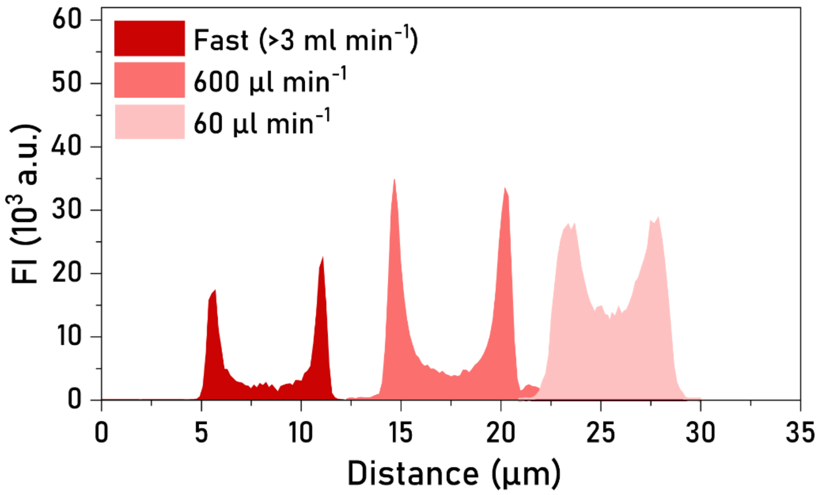

Figure S8. Fluorescence intensity (FI) plot profiles of Texas Red™-lipids distributed across a particle. Overlap of the profiles of a representative particle for ethanol:DPBS gradients produced at >3 ml min^{-1}, 600 μl min^{-1}, or 600 μl min^{-1} buffer addition rate.

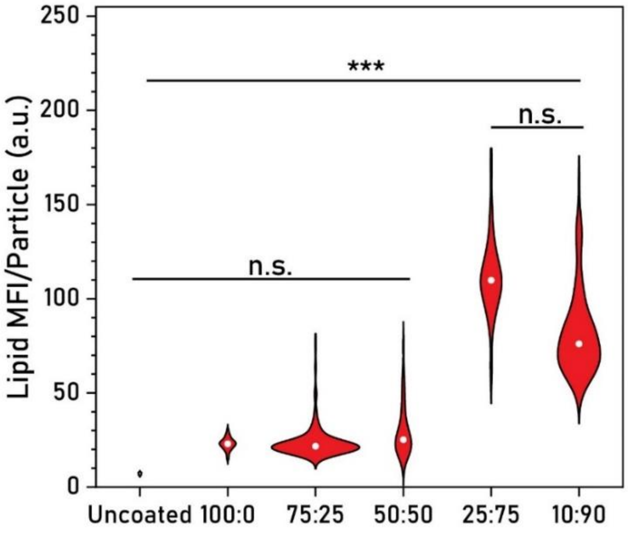

Figure S9. Texas Red™-lipid mean fluorescence intensity (MFI) per particle at different ethanol:DPBS ratios (100:0, 75:25, 50:50, 25:75, and 10:90). Median represented in white dots.

Statistical analysis done through Kruskal-Wallis ANOVA with post hoc Dunn's test for n = 8 (UC), n = 41 (100:0), n = 219 (75:25), n = 40 (50:50), n = 53 (25:75), and n = 106 (10:90) particles.

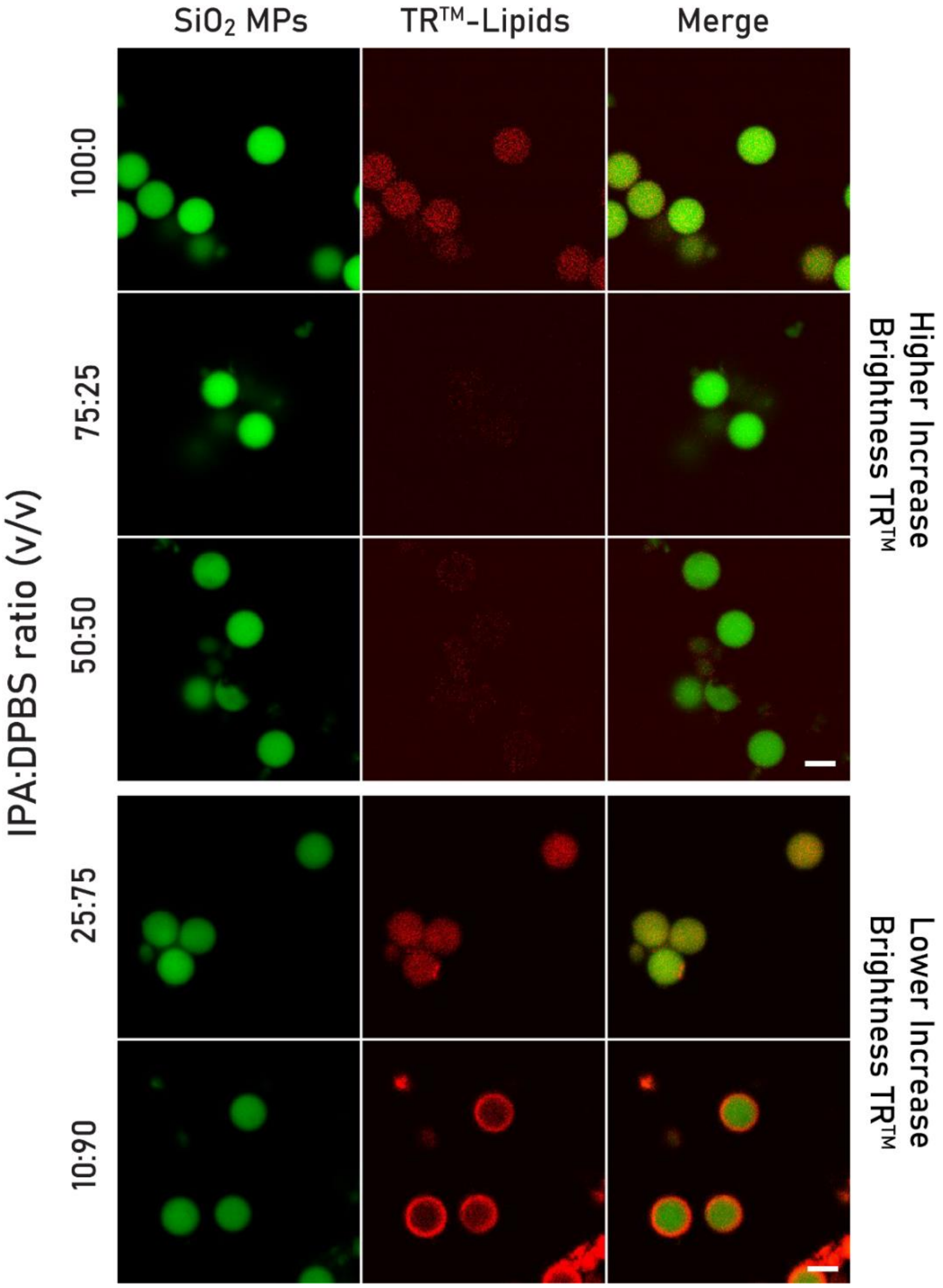

Figure S10. Confocal laser scanning micrographs of coating formation on an IPA:DPBS gradient, obtained from a lipid solution of 0.25 mg ml^{-1} at a fast buffer addition rate (> 3 ml min^{-1}) and recorded at different solvent-to-buffer ratios (100:0, 75:25, 50:50, 25:75, 10:90).

SiO_2 MPs represented in green and Texas Red™-lipids represented in red. Images equally adjusted for brightness, rendering a comparable grayscale within the subsets of 100:0-75:25-50:50 and 25:75-10:90. This adjustment was done in view of improving the visualization of lipid assembly at the early stages of the gradient. Scale bars equal 5 µm.

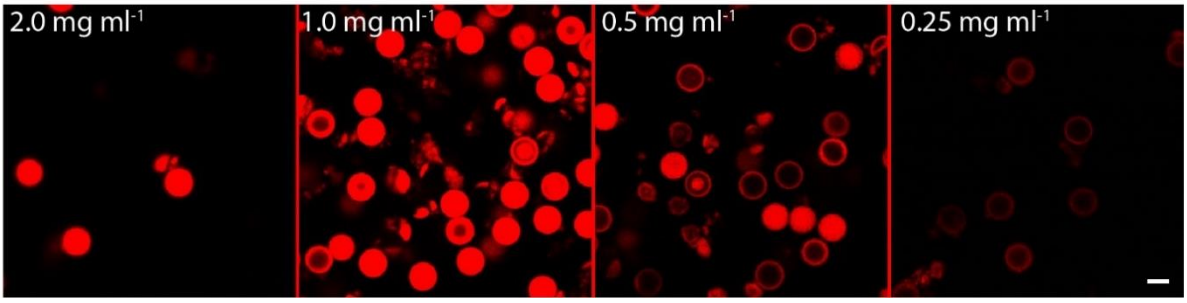

Figure S11. Lipid coverage and infiltration on SiO_2 MPs at 2.0, 1.0, 0.5 and 0.25 mg mL^{-1} lipids, corresponding to a theoretical $n \approx 44$, 88, 176, and 353 lipid bilayers per particle. Coatings obtained at an IPA:DPBS solvent-buffer ratio of 10:90 (v/v) and a fast buffer addition rate (> 3 ml min^{-1}). Green channel omitted for easier visualization of lipid infiltration. Images equally enhanced for brightness (comparable greyscale) to allow the comparison of all formulations. Scale bar equals 5 µm.

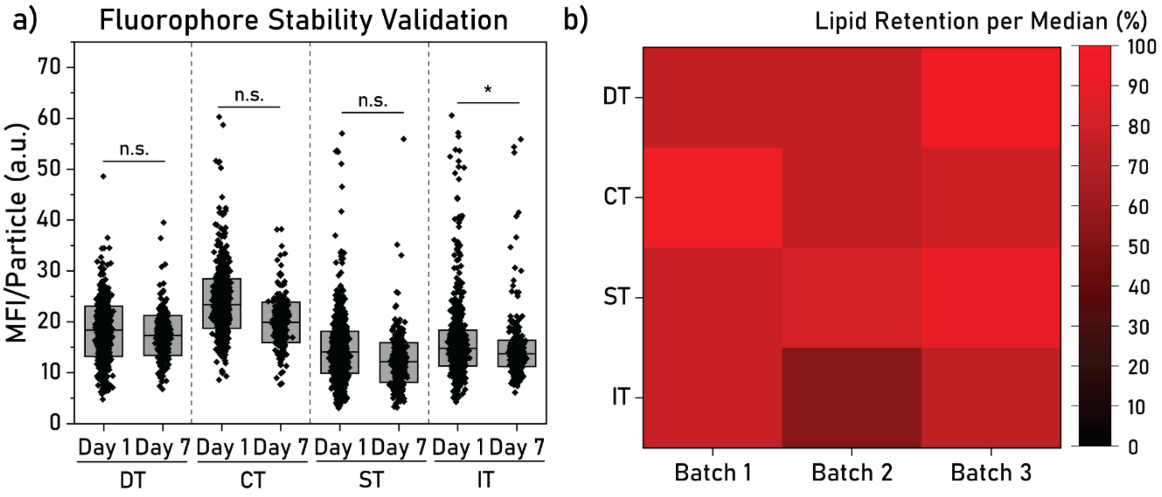

Figure S12. a. Independent fluorophore stability validation study, prior to determining coating stability. The mean fluorescence intensity (MFI) of Texas Red^{TM}-DHPE per particle was compared at days 1 and 7 to determine whether the fluorophore was stable over time. The box indicates the 10-90 percentile, the line represents the median. Statistical analysis made with Mann-Whitney test for a minimal n = 433 (DT), 394 (CT), 548 (ST), and 608 (IT). **b.** Lipid retention percentage, calculated based on the comparison of the median MFI per particle, per formulation, per batch, at days 1 and 7.

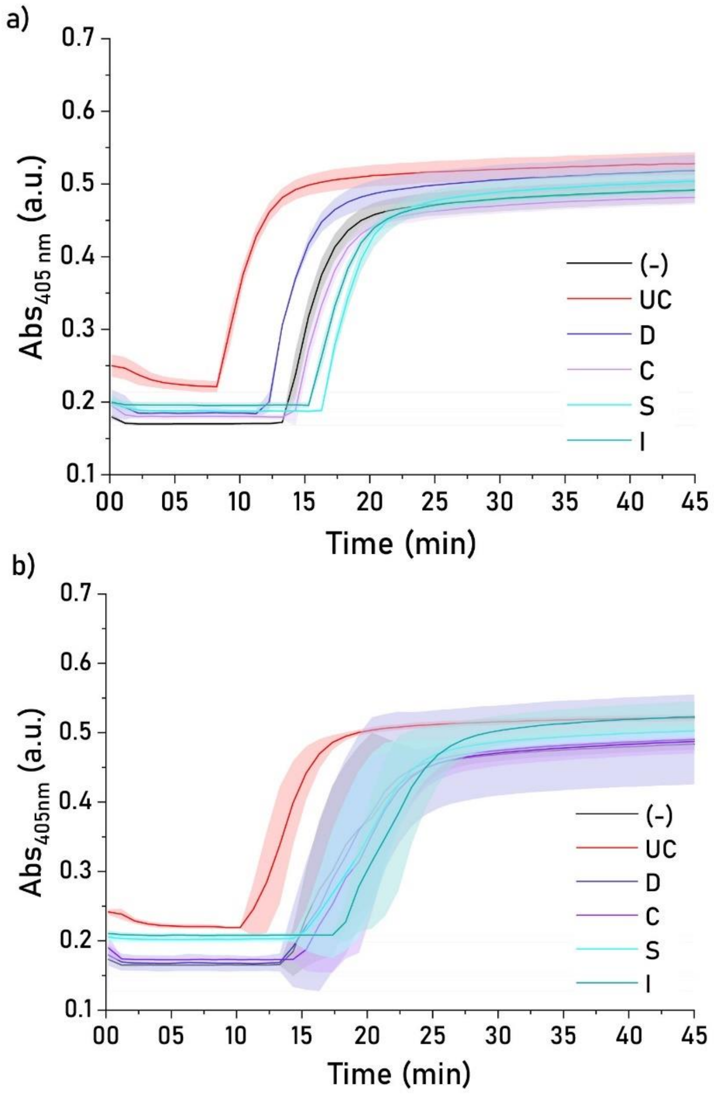

Figure S13. Plasma coagulation curves from fibrin clot formation kinetics. Plasma incubated with buffer (-), uncoated SiO_2 NPs (UC), or coated SiO_2 NPs with formulations D, C, S, and I. Data collected from a second (**a**) and a third batch (**b**), up to a total of n = 3 assays. Third batch represented in main text. Error curves represented for a minimum of three replicate measurements.

Methods S1

To determine the minimum amount of lipids necessary to coat SiO_2 NPs, we first calculated the number of lipids needed to form n lipid bilayers on a single SiO_2 NP, based on a method described elsewhere.[1] Assuming an average diameter (d) of 0.1 µm, we calculated the surface area (A_{NP}) to be 3.14×10^4 nm^2, based on Equation S1:

$$A_{NP} = 4\pi(\frac{d}{2})^2 \qquad \text{(S1)}$$

Knowing that the headgroup area of a single DOPC lipid (A_{DOPC}) is 7.25×10^{-1} nm^{2,} from ref.[2], we estimated that the minimum number of DOPC molecules (N) needed to reach n = 4, 8, 16, or 32 lipid bilayer coatings were, respectively, ca. 3.5×10^5, 7×10^5, 1.4×10^6, and 2.8×10^6, based on Equation S2:

$$N = 2n\frac{A_{NP}}{A_{DOPC}} \qquad \text{(S2)}$$

The choice of n = 4, 8, 16, or 32 was based on a previous study, wherein DOPC lipid bilayer-to-nanoparticle ratios of 2:1, 4:1, and 8:1[1]. Here, the 8:1 ratio was shown to produce the least polydisperse lipid-coated particle suspensions[1], leading to our selection of n between 4 and 32.

Considering that the density of SiO_2 NPs (ρ) is 2.0 g cm^{-3} and the molecular weight (M_W) of DOPC is 786 g mol^{-1}, we then determined that the minimum mass of DOPC per mg of particle ($MR_{DOPC/NP}$) for n = 4, 8, 16, and 32 bilayers were, respectively, 0.43, 0.86, 1.73, and 3.44 mg, based on Equation S3:

$$MR_{DOPC/NP} = \frac{mass\ DOPC}{mass\ NP} = \frac{M_w\ N}{N_{Avogadro} \cdot \frac{4}{3}\pi(\frac{d}{2})^3\rho} \qquad \text{(S3)}$$

For all coating formation tests, we fixed the mass of SiO_2 NPs at 0.5 mg. As such, the lipid masses necessary to achieve $n \approx$ 4, 8, 16, and 32 lipid bilayers on this mass of particles were

0.22, 0.43, 0.86, and 1.73 mg, respectively. Starting with a solvent phase of 500 µl (50 µl SiO_2 NPs + 450 µl lipid), we calculated that lipid solutions should be prepared at 0.48, 0.96, 1.92, and 3.84 mg ml^{-1}. For practicality, we prepared the lipid solutions at 0.5, 1.0, 2.0, and 4.0 mg ml^{-1}. These solutions were subsequently used to produce lipid coatings at a calculated coverage of 4, 8, 16, and 32 lipid bilayers per particle, respectively, henceforth referred to as conditions 4:1, 8:1, 16:1, and 32:1.

Methods S2

To determine the minimum amount of lipids necessary to coat SiO_2 MPs, we used the same methodology as SiO_2 NPs. We had observed that SiO_2 NPs required a lipid concentration of 8:1 lipid bilayers per particle to form a colloidally stable particle suspension, which corresponded to 0.9 mg of lipid per mg of SiO_2 NPs. However, SiO_2 MPs have a significantly lower surface area-to-volume ratio than SiO_2 NPs, which correspond to a much lower lipid concentration needed to achieve the same surface coverage.

To achieve an approximate lipid coverage (i.e., a lipid bilayer-to-particle ratio within the same order of magnitude), we adapted the setup conditions of particle mass, volume, and lipid concentration to 0.4 mg of microparticles, 40 µl of solvent, and 160 µl of lipid solution at 0.25, 0.5, 1.0, and 2.0 mg ml^{-1}. Using the same equations as **Supplementary Method 1**, we determined that these new lipid concentrations corresponded to 0.1, 0.2, 0.4, and 0.8 mg per mg of SiO_2 MPs, or $n \approx 44$, 88, 176, and 353 lipid bilayers per SiO_2 MP, respectively.

References SI

1 Wan, F.; Nylander, T.; Klodzinska S.N.; Foged, C.; Yang, M.; Baldursdottir, S.G; Nielsen, H.M. Lipid Shell-Enveloped Polymeric Nanoparticles with High Integrity of Lipid Shells Improve Mucus Penetration and Interaction with Cystic Fibrosis-Related Bacterial Biofilms. *ACS Applied Materials & Interfaces 2018 10 (13), 10678-10687. doi: 10.1021/acsami.7b19762*

2 Petrache, H.I.; Tristram-Nagle, S.; Gawrisch, K.; Harries, D.; Parsegian, V.A.; Nagle, J.F. Structure and fluctuations of charged phosphatidylserine bilayers in the absence of salt. *Biophys J. 2004 Mar;86(3):1574-86. doi: 10.1016/S0006-3495(04)74225-3.*
